# The mTORC1 complex in pre-osteoblasts regulates whole-body energy metabolism independently of osteocalcin

**DOI:** 10.1038/s41413-020-00123-z

**Published:** 2021-02-08

**Authors:** Pawanrat Tangseefa, Sally K. Martin, Peck Yin Chin, James Breen, Chui Yan Mah, Paul A. Baldock, Gary A. Wittert, Amanda J. Page, Christopher G. Proud, Stephen Fitter, Andrew C. W. Zannettino

**Affiliations:** 1 Adelaide Medical School, Faculty of Health and Medical Science, University of Adelaide, Adelaide, 5005 SA Australia; 2 Cancer Program, Precision Medicine Theme, South Australian Health and Medical Research Institute, Adelaide, 5000 SA Australia; 3 Research Centre for Reproductive Health, School of Paediatrics and Reproductive Health, University of Adelaide, Adelaide, 5005 SA Australia; 4 Computational & Systems Biology program, Precision Medicine Theme, South Australian Health and Medical Research Institute, Adelaide, 5000 SA Australia; 5 Robinson Research Institute, University of Adelaide, Adelaide, SA 5005 Australia; 6 Bioinformatics Hub, University of Adelaide, Adelaide, SA 5005 Australia; 7 Freemasons Foundation Centre for Men’s Health, University of Adelaide, Adelaide, 5005 SA Australia; 8 Skeletal Metabolism Group, Garvan Institute of Medical Research, Sydney, 2010 NSW Australia; 9 Nutrition, Diabetes & Metabolism Program, Lifelong Health Theme, South Australian Health and Medical Research Institute, Adelaide, 5000 SA Australia; 10 School of Biological Sciences, University of Adelaide, Adelaide, 5005 SA Australia; 11 Central Adelaide Local Health Network, Adelaide, SA Australia

**Keywords:** Homeostasis, Diabetes

## Abstract

Overnutrition causes hyperactivation of mTORC1-dependent negative feedback loops leading to the downregulation of insulin signaling and development of insulin resistance. In osteoblasts (OBs), insulin signaling plays a crucial role in the control of systemic glucose homeostasis. We utilized mice with conditional deletion of *Rptor* to investigate how the loss of mTORC1 function in OB affects glucose metabolism under normal and overnutrition dietary states. Compared to the controls, chow-fed *Rptor*_*ob*_^*−/−*^ mice had substantially less fat mass and exhibited adipocyte hyperplasia. Remarkably, upon feeding with high-fat diet, mice with pre- and post-natal deletion of *Rptor* in OBs were protected from diet-induced obesity and exhibited improved glucose metabolism with lower fasting glucose and insulin levels, increased glucose tolerance and insulin sensitivity. This leanness and resistance to weight gain was not attributable to changes in food intake, physical activity or lipid absorption but instead was due to increased energy expenditure and greater whole-body substrate flexibility. RNA-seq revealed an increase in glycolysis and skeletal insulin signaling pathways, which correlated with the potentiation of insulin signaling and increased insulin-dependent glucose uptake in *Rptor*-knockout osteoblasts. Collectively, these findings point to a critical role for the mTORC1 complex in the skeletal regulation of whole-body glucose metabolism and the skeletal development of insulin resistance.

## Introduction

An appropriate supply of glucose is fundamental for efficient cellular function and requires complex inter-tissue communication networks. The skeleton plays an important role in the regulation of systemic glucose metabolism, with deletion of the insulin receptor (INSR) in the bone-forming osteoblast (OB) (INSR_OB_^*−/−*^ mice) leading to elevated blood glucose, reduced serum insulin, and insulin resistance.^1,2^ Conversely, mice with OB-specific deletion of either FoxO1 or ATF4, negative regulators of insulin signaling, display the opposite phenotype to INSR_OB_^−/−^ mice with improved glucose disposal and insulin sensitivity.^3,4^ Apart from hepatocytes, OBs are the only cell type in which disruption of insulin signaling hampers glucose metabolism in mice fed a normal chow diet (NCD),^1^ further highlighting the importance of the skeleton in regulating glucose homeostasis.

Mechanistically, insulin signaling in OBs regulates whole-body glucose metabolism via a feed forward loop involving the bone-specific protein osteocalcin (OCN). Undercarboxylated OCN (unOCN) acts as a hormone that improves glucose handling by directly stimulating pancreatic β-cell proliferation^5^ and insulin secretion^6^ and indirectly stimulating glucagon-like peptide-1 secretion by the gut.^7^ unOCN also increases insulin sensitivity in liver, muscle and adipose tissue by increasing adiponectin,^6,8^ an adipokine that modulates glucose homeostasis independently of insulin. Consistent with these observations, daily administration of OCN to insulin-resistant high-fat diet (HFD)-fed mice partially restores their insulin sensitivity and glucose tolerance.^8,9^

The mammalian target of rapamycin complex 1 (mTORC1) is a critical mediator of insulin signaling and a primary nutrient-sensing pathway that coordinates anabolic and catabolic processes to control cellular growth and metabolism. Insulin signaling is negatively regulated by mTORC1, which becomes hyper-activated in response to nutrient overload leading to an insulin-resistant state (review in^10^). Accumulating evidence suggests that in addition to being vital for insulin action at an intracellular level, mTORC1 plays an important role in controlling whole-body glucose metabolism. Indeed, conditional deletion of *Rptor* (encoding RAPTOR, an essential component of mTORC1) in insulin-responsive tissues results in systemic alterations in metabolism, albeit in a tissue-specific manner.^11–13^

Previous loss-of-function studies by our group^14^ and others^15,16^ have revealed an important role for OB-mTORC1 in the control of pre- and post-natal skeletal development. Loss of mTORC1 function in pre-OBs (*Rptor*_*ob*_^*−/−*^ mice) results in osteopenia and skeletal fragility due, in part, to a decrease in the protein synthetic properties of OBs.^14^ Conversely, mice with OB-specific deletion of *Tsc2*, a negative regulator of mTORC1 (∆Tsc2 mice), exhibit increased bone mass and accumulation of poorly differentiated OBs.^17^ With advanced age, ∆Tsc2 mice develop a metabolic phenotype similar to INSR_OB_^*−/−*^ mice despite having high levels of circulating OCN.^17^ Collectively, these data suggest a potential role for the mTORC1 complex in osteoblasts (OB-mTORC1) in the skeletal regulation of glucose metabolism.

To investigate this question, we examined the metabolic phenotype of *Rptor*_*ob*_^*−/−*^ mice under both NCD and HFD conditions and found that OB-specific deletion of *Rptor* has a profound effect on whole-body metabolism, as evidenced by reduced fat mass, increased insulin sensitivity and glucose disposal and elevated serum adiponectin levels. Moreover, both pre- and post-natal deletion of *Rptor* in osteoprogenitor cells led to protection from HFD-induced weight gain and metabolic dysfunction supporting a central role for the mTORC1 complex in bone in the development of systemic insulin resistance.

## Results

### Loss of OB-mTORC1 function results in lean mice with increased energy expenditure and a metabolic shift towards fat oxidation

Mice with conditional deletion of *Rptor* in osteoprogenitor cells were generated as previously described^14^ and owing to previously observed gene dosage effects in these mice,^14,18^ both heterozygous (*Rptor*_*ob*_^*+/*^^−^) and homozygous (*Rptor*_*ob*_^*−/−*^) knockout animals analyzed. As our preliminary studies revealed that body weight adjusted fat mass, lean mass adjusted total energy expenditure (TEE), serum OCN levels (both total and undercarboxylated), fasting glucose levels and glucose metabolism (glucose tolerance and insulin sensitivity) are unaltered in *Osx1-GFP:Cre* mice, relative to age/sex matched wild-type mice, wild-type littermates were used as the control group (Supplementary Fig. 1a–n). It is also important to note that we failed to detect Cre-mediated deletion of *Rptor* in iWAT and no differences in RAPTOR protein levels in metabolic organs including adipose tissue, muscle, or liver was observed in heterozygous or homozygous knockout animals, relative to controls, suggesting no off-target *Rptor* deletion (Supplementary Fig. 2a–c). Furthermore, no detectable off-target *Cre* recombinase activity in metabolic tissues including spleen, liver, brain, and intestine has previously been observed in *Osx1-GFP:Cre* mice.^19^

**Fig. 1:**
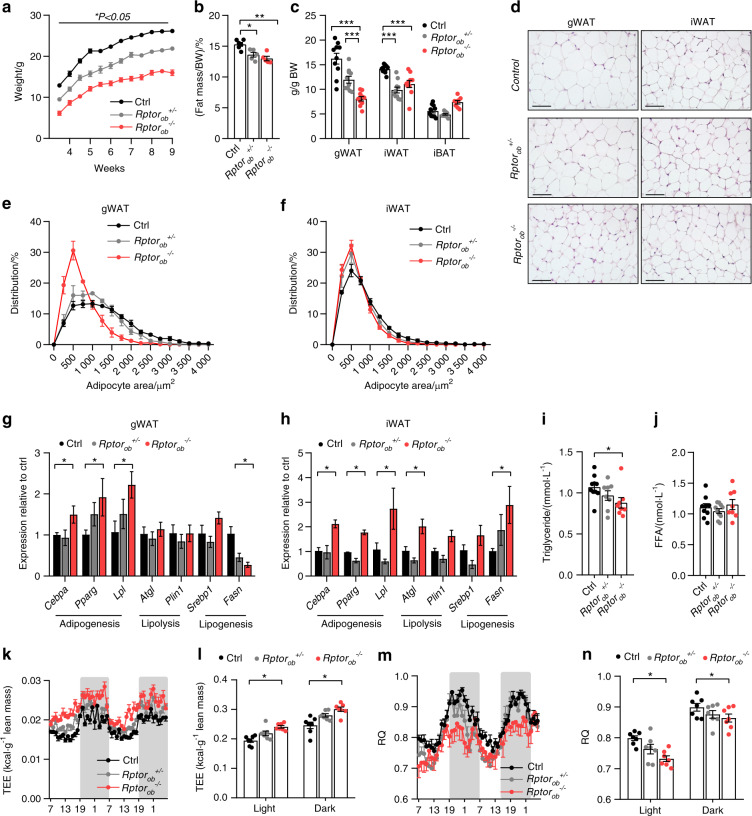
Loss of mTORC1 function in pre-OBs results in a lean phenotype. **a** Temporal change in body weight (*n* ≥ 15/genotype). **b** %Fat normalized to total body weight. **c** Body weight-adjusted gonadal and inguinal white adipose tissue (gWAT and iWAT) and interscapular brown adipose tissue (iBAT) mass (9 weeks of age; *n* = 10/group). **d** Representative H&E section of gWAT and iWAT, scale bar = 100 µm. **e**, **f** Size distribution of gWAT and iWAT adipocytes calculated from H&E stained sections using Image J (*n* = 5 sections/mouse, *n* = 3–6/genotype). **g**, **h** Gene expression levels of adipogenesis, lipogenesis, and lipolysis genes, normalized to *Actb*, in gWAT and iWAT (*n* = 3–5/genotype). **i** Serum triglyceride levels (*n* = 8–10/genotype). **j** Serum-free fatty acid levels (*n* = 8–10/genotype). **k** Total energy expenditure, normalized to lean mass, over 48 h and **l** per light/dark cycle. **m** Average respiratory quotient (RQ) over 48 h and **n** per light/dark cycle. Data are expressed as mean ± SEM from *n* = 7/group, unless indicated otherwise. *P < 0.05, **P < 0.01, ***P < 0.001, one-way ANOVA with Tukey’s post hoc test. **k**, **m** Shaded regions represent dark cycle

From weaning, NCD-fed *Rptor*_*ob*_^−/−^ mice weighed significantly less than control and *Rptor*_*ob*_^*+/−*^ littermates (Fig. 1a). An assessment of body composition by dual X-ray absorptometer (DXA) revealed a significant reduction in %fat mass and body weight-adjusted white adipose tissue (WAT) depots in both *Rptor*_*ob*_^*+/−*^ and *Rptor*_*ob*_^*−/−*^ mice, relative to controls (Fig. 1b, c), while %lean mass and normalized weights of major lean organs were unchanged (Supplementary Fig. 3a, b). Histological examination of WAT depots revealed a significant shift toward adipocyte hypotrophy in gonadal and inguinal WAT (gWAT and iWAT, respectively) in *Rptor*_*ob*_^*−/−*^ mice compared to the controls (Fig. 1d–f). Consistent with this, the expression levels of key adipogenic genes (*Pparg*, *Cebpa*, and *Lpl*) were significantly increased in fat depots of *Rptor*_*ob*_^*−/−*^ mice (Fig. 1g, h). The levels of *Fasn* mRNA, a regulator of lipogenesis, was decreased in gWAT but increased in iWAT. In contrast, the expression of both lipolytic genes (*Atgl* and *Plin*) was increased and unchanged in iWAT and gWAT, respectively, in *Rptor*_*ob*_^*−/−*^ mice compared to the control (Fig. 1g, h).

The reduced adiposity of *Rptor*_*ob*_^*+/−*^ and *Rptor*_*ob*_^*−/−*^ mice was independent of changes in food intake (despite reduced leptin levels (Supplementary Fig. 3c)), total physical activity (XYZ counts) or percentage of fecal lipid content (Supplementary Fig. 3d–g). To account for genotypic differences in body weight, measurements of food intake, oxygen consumption (VO_2_), carbon dioxide production (VCO_2_), and TEE data were normalized to lean mass. TEE was significantly increased in *Rptor*_*ob*_^*−/−*^ mice during both the light and dark periods (Fig. 1k, l). Furthermore, *Rptor*_*ob*_^*−/−*^ mice exhibited a significantly higher rate of VO_2,_ with transiently higher rate of VCO_2_ (Supplementary Fig. 3h-k). Consequently, the respiratory quotient values (RQ, Fig. 1m, n) were significantly lower in *Rptor*_*ob*_^*−/−*^ mice compared to the controls, suggesting a preference for fat oxidation. The elevated TEE and lower RQ observed in the *Rptor*_*ob*_^*−/−*^ mice were associated with an increase in expression of *Pcg1a* and *Ucp1* in interscapular brown adipose tissue (iBAT) (Supplementary Fig. 3l). However, no consistent changes in iBAT UCP1 protein levels were observed suggesting the elevated TEE and lower RQ was not due to increased iBAT thermogenesis (Supplementary Fig. 3m). Measurements of circulating triglyceride (TG) and free fatty acids (FFA) levels revealed a significantly lower TG levels in the serum of *Rptor*_*ob*_^*−/−*^ mice compared to controls, whereas no significant difference was observed in levels of FFAs (Fig. 1i, j).

### *Rptor*_*ob*_^*−/−*^ mice display improved glucose metabolism and increased insulin sensitivity

**Fig. 2:**
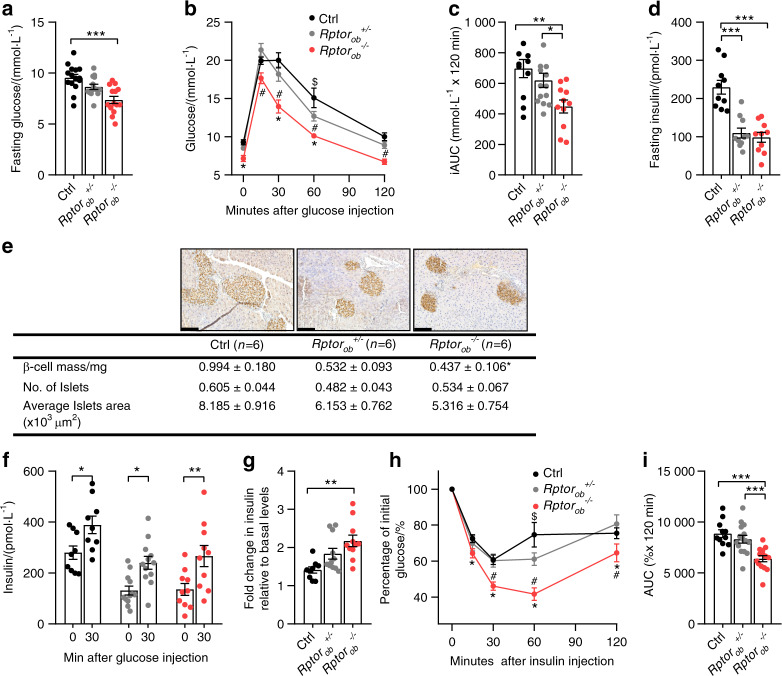
NCD-fed *Rptor*_*ob*_^*−/−*^ mice display an improvement in glucose metabolism. **a** Fasting blood glucose levels in 9-week-old mice (*n* = 15/genotype). **b** GTT blood glucose levels in 9-week-old mice, and **c** incremental area under the curve analysis (*n* = 11–14/genotype). **d** Fasting serum insulin levels (*n* = 10/genotype). **e** Representative images of insulin immunostaining in pancreas samples from 9-week-old mice. Scale bars = 100 µm and histomorphometric analysis of β-cell area, islet number, and islet size (*n* = 6/genotype). **f** Glucose-stimulated insulin levels and **g** fold change in insulin levels at 30 min relative to basal levels (*n* = 9–12/genotype). **h** ITT blood glucose levels in 8-week-old mice and **i** area under the curve analysis (*n* = 11–15/group). All panels except **b** and **h**: data are expressed as mean ± SEM. *P < 0.05, **P < 0.01, ***P < 0.001, one-way ANOVA with Tukey’s post hoc test. **b**, **h** *P < 0.05 between *Rptor*_*ob*_^*−/−*^ and Ctrl, ^#^*P* < 0.05 between *Rptor*_*ob*_^*−/−*^ and *Rptor*_*ob*_^*+/−*^ and ^$^*P* < 0.05 between *Rptor*_*ob*_^*+/*^^−^ and Ctrl, two-way ANOVA with Tukey’s post hoc test

Fasting glucose levels were significantly lower in *Rptor*_*ob*_^*−/−*^ mice (−22.8% and −14.9% compared to control and *Rptor*_*ob*_^*+/−*^ mice, respectively, Fig. 2a), while no difference was observed between *Rptor*_*ob*_^*+/−*^ and control mice. When fasted animals were challenged with a bolus of glucose, *Rptor*_*ob*_^*−/−*^ mice exhibited enhanced glucose clearance compared to controls, whereas no change in glucose tolerance was observed in *Rptor*_*ob*_^*+/−*^ mice (Fig. 2b, c). Hypoglycemia was not due to hyperinsulinemia, as fasting insulin levels were significantly reduced in both *Rptor*_*ob*_^*−/−*^ and *Rptor*_*ob*_^*+/−*^ mice compared to controls (Fig. 2d). Consistent with lower circulating insulin levels, histological analysis of pancreatic tissue revealed a significant decrease in the β-cell mass and a trend toward a significant decrease in average islet area (*P* = 0.061) in *Rptor*_*ob*_^*−/−*^ mice, with no significant change in pancreatic islet number (Fig. 2e). Despite their reduced insulin levels, the ability to secrete insulin in response to a glucose bolus [glucose-stimulated insulin secretion (GSIS) tests] in both *Rptor*_*ob*_^*+/−*^ and *Rptor*_*ob*_^*−/−*^ mice remained functional as demonstrated by a significant increase in insulin levels at 30 min post glucose bolus compared to the basal levels (Fig. 2f). Moreover, the relative insulin levels at 30 min (i.e., insulin fold induction relative to basal levels to account for lower basal insulin levels) in response to glucose bolus were significantly higher in *Rptor*_*ob*_^*+/−*^ and *Rptor*_*ob*_^*−/−*^ mice relative to controls (Fig. 2g).

The reduced fasting insulin and glucose levels observed in *Rptor*_*ob*_^*−/−*^ mice, coupled with their improved glucose disposal and ability to maintain lower levels of circulating insulin after glucose bolus, suggest that these mice were more sensitive to insulin compared to their *Rptor*_*ob*_^*+/−*^ and control littermates. As expected, insulin tolerance tests (ITTs) indicated a significant increase in insulin sensitivity in *Rptor*_*ob*_^*−/−*^ mice compared to both controls and *Rptor*_*ob*_^*+/−*^ mice, while no difference was observed in *Rptor*_*ob*_^*+/−*^ mice relative to controls (Fig. 2h, i). Of note, an increased sensitivity to insulin was not attributable to increased insulin sensitivity in muscle and liver, as evidenced by the lack of changes in gene expression for markers of insulin sensitivity (*Pcg1α* and its target genes, *Mcad* and *Nrf1*, in muscle and *Foxa2* in liver: Supplementary Fig. 4a, b). No significant changes in the expression levels of gluconeogenesis genes, *Pepck* and *G6p*, was observed (*P* = 0.55 and *P* = 0.57, respectively, controls vs. *Rptor*_*ob*_^*−/−*^ mice). Conversely, we found that the expression levels of glucokinase, the first-rate limiting enzyme in glycolysis, was upregulated in the *Rptor*_*ob*_^*−/−*^ mice (Supplementary Fig. 4a). Moreover, no significant difference in insulin-stimulated AKT phosphorylation was observed in muscle and liver tissue lysates isolated from *Rptor*_*ob*_^*−/−*^ mice relative to control mice (Supplementary Fig. 4c, d).

### The metabolic improvements observed in *Rptor*_*ob*_^*−/−*^ mice occur independently of osteocalcin

**Fig. 3:**
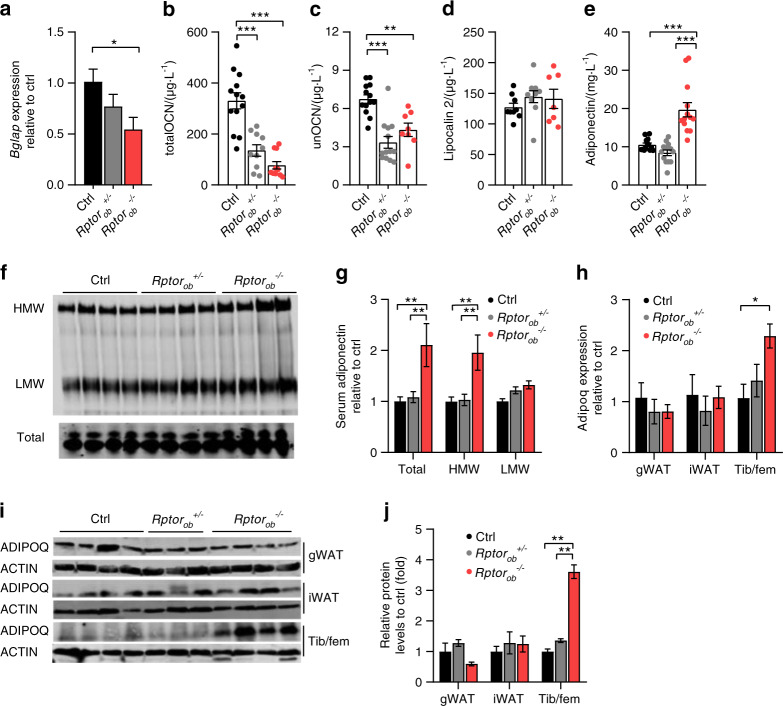
Improvements in glucose metabolism observed in NCD-fed *Rptor*_*ob*_^*−/−*^ mice occur independently of osteocalcin. **a** *Bglap* gene expression, normalized to *Actb*, in flushed long bones (femur/tibia, *n* = 3–4/genotype) from 9-week-old mice. **b** Serum OCN (total) levels (*n* = 10–13/genotype). **c** Serum unOCN levels (*n* = 8–13/genotype). **d** Serum lipocalcin-2 levels (*n* = 7–10/genotype). **e** Serum adiponectin levels (*n* = 13/genotype). **f**, **g** Western immunoblot analysis and quantification of high molecular weight (HMW), low molecular weight (LMW), and total adiponectin levels in serum (*n* = 4/genotype). **h** *Adipoq* gene expression, normalized to *Actb*, in gWAT, iWAT, and whole long bone (tibia/femur) samples (*n* = 3–5/genotype). **i** Levels of total adiponectin protein expression in gWAT, iWAT, and tibia/femur samples (*n* = 3–4/genotype) and **j** Quantitative analysis of adiponectin protein levels, relative to ACTIN, from **i**. Data are expressed as mean ± SEM. *P < 0.05, **P < 0.01, ***P < 0.001, one-way ANOVA with Tukey’s post hoc test

As shown in Fig. 3a–c, OCN gene (*Bglap*) expression and circulating levels of total and unOCN were significantly reduced in *Rptor*_*ob*_^*+/−*^ and *Rptor*_*ob*_^*−/−*^ mice compared to controls (consistent with the previously reported osteopenia observed in these mice^14^), suggesting that OCN is unlikely to be mediating the metabolic improvements observed in *Rptor*_*ob*_^*−/−*^ mice. In addition to OCN, more recent studies have shown that the skeleton can regulate appetite and glucose homeostasis via lipocalin-2 (LCN2),^20^ an OB-enriched, secreted protein. To determine if increased LCN2 levels may be compensating for the decreased unOCN levels observed in *Rptor*_*ob*_^*+/−*^ and *Rptor*_*ob*_^*−/−*^ mice, serum LCN2 levels were measured; however, no differences were found across the three genotypes (Fig. 3d).

In contrast, the increase in insulin sensitivity in *Rptor*_*ob*_^*−/−*^ mice was associated with a significant (Approximately twofold) elevation in circulating adiponectin levels (Fig. 3e). In particular, levels of the high molecular weight (HMW) form of adiponectin, the most bio-active form which negatively correlates with insulin resistance,^21^ were significantly higher in *Rptor*_*ob*_^*−/−*^ mice (Fig. 3f, g). Analysis of adiponectin mRNA (*Adipoq*) and protein levels revealed WAT (iWAT and gWAT) was not the source (Fig. 3h–j). In addition to WAT, a distinct population of adipocytes that reside within the bone marrow adipose tissue (BMAT) has also been shown to contribute to hyperadiponectinemia,^22^ and we have previously reported markedly elevated BMAT levels in *Rptor*_*ob*_^*−/−*^ mice.^14^ Consistent with an increase in BMAT, levels of *Adipoq* mRNA were increased (~2.3-fold) in long bones (combined Tibia/femur: Tib/fem) from *Rptor*_*ob*_^*−/−*^ mice, and a ~2.5-fold increase in adiponectin protein levels was detected by western blot (Fig. 3h–j).

### *Rptor*_*ob*_^*−/−*^ mice are protected from diet-induced obesity and insulin resistance

**Fig. 4:**
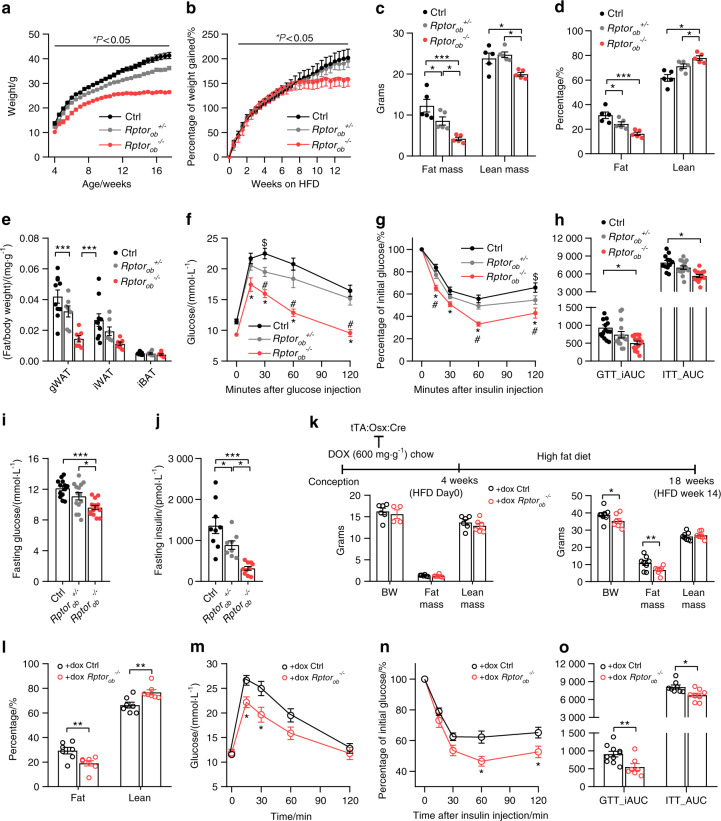
Pre- and post-natal deletion of *Rptor* protects against high-fat diet-induced obesity and insulin resistance. **a** Temporal change in body weight (*n* ≥ 15/genotype). **b** % of body weight gained in response to high-fat diet (HFD). **c** Body analysis by DXA scanning of total mass and **d** composition as percentage of body weight in 18-week-old HFD-fed mice (*n* = 5/genotype). **e** Body weight adjusted fat depot (*n* = 7–10/genotype). **f** GTT blood glucose levels in 17-week-old mice. **g** ITT blood glucose levels in 16-week-old mice. **h** Incremental AUC analysis of GTT from **f** and AUC analysis of ITT from **g** (*n* = 12–15/genotype). **i** Fasting blood glucose levels (*n* = 13/genotype). **j** Fasting serum insulin levels (*n* = 8–10/genotype). **k** Schematic showing use of doxycycline (DOX) administration to suppress the tTA:Osx:cre transgene until the pups are 4 weeks of age. DOX chow was replaced with a high-fat diet to induce obesity and insulin resistance *(upper panel)*. Body composition at 4 weeks (HFD day 0; *n* = 4–6/genotype) and 18 weeks (HFD week 14; *n* = 7–8/genotype) of age *(lower panels)*. **l** Body composition as percentage of total body weight *n* = 7–8/genotype). **m** GTT blood glucose levels in 17-week-old mice. **n** ITT blood glucose levels. **o** Incremental AUC analysis of GTT from **m** and AUC analysis of ITT from **n** (*n* = 12–15/genotype). Data presented as mean ± SEM. **a**–**e** *P < 0.05, ***P < 0.001, One-way ANOVA with Tukey’s post hoc test. **f**, **g** *P < 0.05 between *Rptor*_*ob*_^*−/−*^ and Ctrl, ^#^*P* < 0.05 between *Rptor*_*ob*_^*−/−*^ and *Rptor*_*ob*_^*+/*^^−^ and ^$^*P* < 0.05 between *Rptor*_*ob*_^*+/−*^ and Ctrl, two-way ANOVA with Tukey’s post hoc test. **k**–**o** *P < 0.05, **P < 0.01, Student’s *t*-test

In light of the metabolic phenotype of NCD-fed *Rptor*_*ob*_^*−/−*^ mice and previous data demonstrating that nutrient overload leads to hyperactivation of mTORC1 signaling and the development of insulin resistance,^23^ we next examined the response of *Rptor*_*ob*_^*−/−*^ mice to an obesogenic diet. Initial studies, performed on 8-week-old animals maintained on HFD from weaning (i.e., ~4 weeks), showed that this timeframe was insufficient to induce insulin resistance in the controls (Supplementary Fig. 5a–d), thus all subsequent HFD studies were performed on 16-week-old mice following 12 weeks of HFD. As shown in Fig. 4a, b, *Rptor*_*ob*_^*−/−*^ mice gained weight at a similar rate to control and *Rptor*_*ob*_^*+/−*^ mice; however, after 6 weeks of HFD, their weight gains plateaued. End-of-study body composition analyses revealed that *Rptor*_*ob*_^*−/−*^ mice remained relatively lean with fat mass 50% and 66% lower compared to control and *Rptor*_*ob*_^*+/−*^ mice, respectively (Fig. 4c). Fat mass (as percentage of body weight) was significantly lower in *Rptor*_*ob*_^*−/−*^ mice whilst the percentage of lean mass was significantly higher in both *Rptor*_*ob*_^*+/−*^ and *Rptor*_*ob*_^*−/−*^ mice compared to the controls (Fig. 4d). Consistent with this, the major fat pads were significantly smaller in *Rptor*_*ob*_^*−/−*^ mice compared to both control and *Rptor*_*ob*_^*+/−*^ mice, whereas no differences in the weights of other lean organs were observed (Fig. 4e and Supplementary Fig. 6a).

Strikingly, the metabolic improvements observed in NCD-fed *Rptor*_*ob*_^*−/−*^ mice were also observed in HFD-fed *Rptor*_*ob*_^*−/−*^ mice. Compared to control and *Rptor*_*ob*_^*+/−*^ mice, *Rptor*_*ob*_^*−/−*^ mice exhibited improved glucose clearance and increased insulin sensitivity (Fig. 4f–h), with these improvements accompanied by a reduction in fasting glucose and insulin levels and a trend toward increased GSIS (*P* = 0.07) (Fig. 4i, j and Supplementary Fig. 6b, c). Moreover, smaller β-cell islet hypertrophy were observed in *Rptor*_*ob*_^*−/−*^ mice (Supplementary Fig. 6d) which is likely to be a secondary effect of persistently low glucose levels.

*Rptor*_*ob*_^*−/−*^ mice have significantly lower body weight adjusted fat mass, under both normal chow and high-fat feeding, which is likely to play an important role in the metabolic phenotype of these mice. The lower %fat mass is potentially a compensatory mechanism as a consequence of deleting *Rptor* during early development (from E13.5^24^). To rule out this possibility, we utilized the tetracycline-repressible element of the tTA:Osx:Cre transgene^24^ to examine the metabolic effects of inhibiting mTORC1 function in skeletally mature mice. Deletion of *Rptor* was suppressed (via doxycycline-supplemented chow diet) until 4 weeks of age and mice were then challenged with a HFD (Fig. 4k). In contrast to the pre-natal *Rptor* deletion model, no differences in body weight and body composition was observed between dox-treated control littermates and dox-treated *Rptor*_*ob*_^*−/−*^ mice (+dox *Rptor*_*ob*_^*−/−*^) at 4 weeks of age (Fig. 4k). However, after 14 weeks on a HFD (at 18 weeks of age), +dox *Rptor*_*ob*_^*−/−*^ mice recapitulated several key protective phenotypes observed in the HFD-fed *Rptor*_*ob*_^*−/−*^ mice including resistance to weight gain, increased glucose tolerance and increased insulin sensitivity (Fig. 4k–o).

**Fig. 5:**
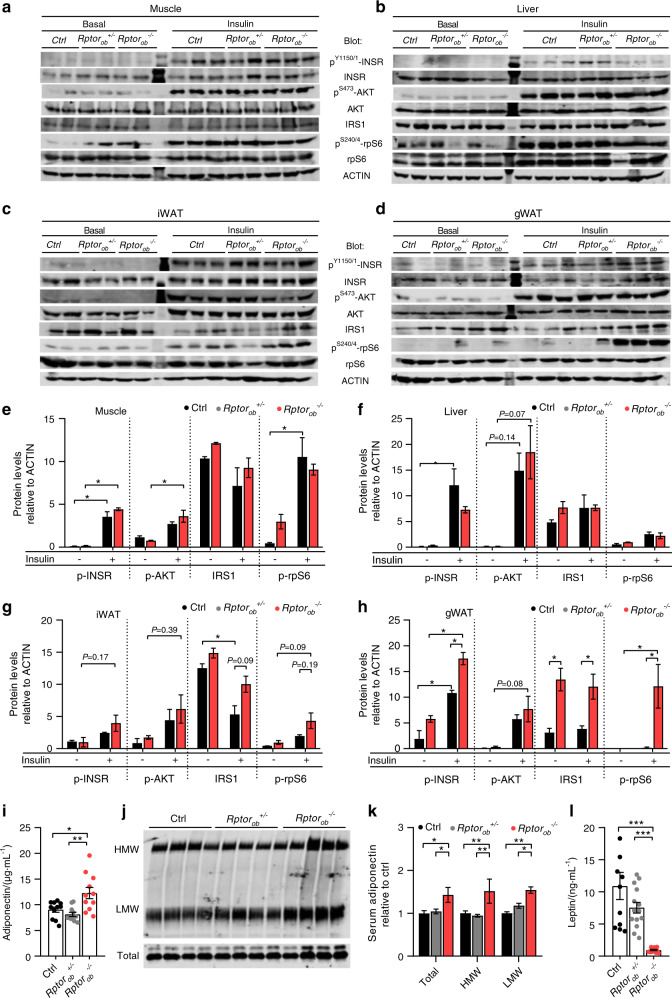
Increased insulin signaling in adipose tissue depots and elevated serum adiponectin in HFD-fed *Rptor*_*ob*_^*−/−*^ mice. Western immunoblot analysis of indicated protein levels in skeletal muscle (**a**), liver (**b**), iWAT (**c**), and gWAT (**d**) tissues from 18-week-old HFD-fed mice collected after a 6-h fast, under either basal (6-h fast + PBS; *n* = 2/genotype) or insulin-stimulated (6-h fast + insulin stimulation; *n* = 2–3/genotype) conditions. **e–h** Quantitative analysis of indicated protein levels from **a**–**d**, relative to ACTIN. **i** Serum adiponectin levels (*n* = 10–15/genotype). **j** Western immunoblot analysis of HMW, LMW forms and total adiponectin levels in sera from 18-week-old HFD-fed mice (*n* = 4/genotype). **k** Quantitative analysis of protein levels from **j**. **l** Serum leptin levels (*n* = 11–14/genotype). All panels except **e**–**h**: data are expressed as mean ± SEM. *P < 0.05, **P < 0.01, ***P < 0.001, one-way ANOVA with Tukey’s post hoc test. **e**–**h** *P* < 0.05, two-way ANOVA with Tukey’s post hoc test

To further investigate insulin sensitivity in *Rptor*_*ob*_^*−/−*^ mice, we examined the acute response to insulin in liver, muscle and adipose tissue. An increase in insulin-stimulated phosphorylation of INSR (p-INSR) was observed in all the tissues examined. Furthermore, significantly higher levels of p-INSR were observed in the gWAT of *Rptor*_*ob*_^*−/−*^ mice compared to the controls (Fig. 5d, h). No significant differences in insulin-stimulated AKT activation was observed between genotypes in these tissues (Fig. 5a–h). We also examined the levels of total IRS1 and the activation status of rpS6 (Ser^240/244^), an effector protein modulated by S6K1, an mTORC1 substrate. mTORC1 negatively regulates insulin signaling leading to phosphorylation of IRS1 and subsequent ubiquitin-mediated degradation.^25,26^ A significant increase in insulin-stimulated rpS6 phosphorylation (p-rpS6) was observed in the gWAT of *Rptor*_*ob*_^*−/−*^ mice relative to controls (Fig. 5d, h). An increase in p-rpS6 was also observed in iWAT of *Rptor*_*ob*_^*−/−*^ mice, however, this did not reach significance (*P* = 0.19) (Fig. 5c, g). A significantly higher level of total IRS1 were observed in the gWAT of *Rptor*_*ob*_^*−/−*^ mice, both basal and insulin-stimulated, compared to controls (Fig. 5d, h) while an increase total IRS1 was also observed in iWAT, under insulin stimulation, however, this did not reach significance (*P* = 0.09) (Fig. 5c, g). Decreased levels of total IRS1 in adipocytes has previously been reported in insulin-resistant subjects^27^ and led to impaired glucose uptake in a type 2 diabetes animal model.^28^ Collectively, these results suggest that an enhanced insulin signaling in the fat depots of *Rptor*_*ob*_^*−/−*^ mice may account for the higher peripheral sensitivity to insulin (Fig. 4g). Consistent with higher insulin sensitivity, serum adiponectin levels (both the HMW and LMW forms) were elevated in HFD-fed *Rptor*_*ob*_^*−/−*^ mice relative to both *Rptor*_*ob*_^*+/−*^ and control mice (Fig. 5i–k) whereas serum leptin levels where significantly lower in *Rptor*_*ob*_^*−/−*^ mice relative to *Rptor*_*ob*_^*+/−*^ and control mice, concordant with reduced fat mass in these mice (Fig. 5l).

### Increased energy expenditure, greater substrate flexibility, and browning of white adipose tissue in *Rptor*_*ob*_^*−/−*^ mice

**Fig. 6:**
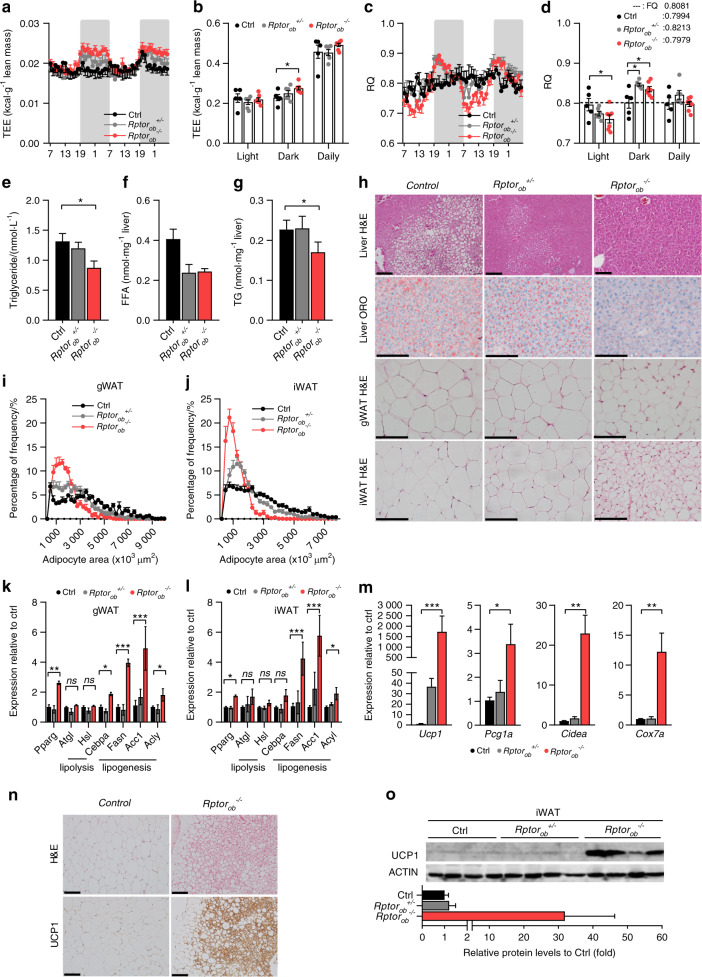
HFD-fed *Rptor*_*ob*_^*−/−*^ mice exhibit greater substrate flexibility and browning of white adipose tissue. **a** Total energy expenditure (TEE), normalized to lean mass, over 48 h and **b** per light/dark cycle. **c** Average RQ over 48 h and **d** per light/dark cycle. **e** Serum triglyceride levels (*n* = 8–10/genotype). **f** Hepatic triglyceride levels and **g** hepatic free fatty acid levels in 18-week-old HFD-fed mice (*n* = 3–4/genotype). **h** Representative images of H&E and Oil Red O-stained sections of liver, gWAT, and iWAT, scale bar = 100 µm. **i**, **j** Size distribution of gWAT and iWAT adipocytes calculated from H&E stained sections using Image J (*n* = 5 sections/mouse, *n* = 5–6/genotype). **k**, **l** Gene expression levels of adipogenesis, de novo lipogenesis, and lipolysis markers in gWAT and iWAT tissues, normalized to *Actb* (*n* = 3–5/genotype). **m** Gene expression levels of brown adipose tissue markers in iWAT tissue, normalized to *Actb* (*n* = 3–5/genotype). **n** Representative images of H&E and UCP1 immunostaining section of iWAT from HFD-fed mice, scale bar = 100 µm. **o** Levels of UCP1 protein expression in iWAT (*top*) and quantitative analysis of protein levels relative to β-actin (*bottom*) (*n* = 4/genotype). Data are expressed as mean ± SEM. *P < 0.05, **P < 0.01, ***P < 0.001, one-way ANOVA with Tukey’s post hoc test

As shown in Fig. 6, TEE in HFD-fed *Rptor*_*ob*_^*−/−*^ mice remained similar to control and *Rptor*_*ob*_^*+/−*^ mice during the light cycle but was significantly higher during the dark cycle, while food intake, total physical activity and fecal lipid content were equivalent across all genotypes (Fig. 6a, b and Supplementary Fig. 7a–e). Interestingly, while 24-h RQ values were similar, the hourly RQ patterns were noticeably different (Fig. 6c, d). To interrogate the hourly RQ patterns further, we calculated the food quotient (FQ), where FQ is the theoretical RQ produced by the diet^29^ and 24-h RQ ≈ FQ indicates a state of energy and macronutrient balance (for the HFD diet used herein, FQ = 0.8081; Supplementary Fig. 7f). Both *Rptor*_*ob*_^*−/−*^ and *Rptor*_*ob*_^*+/−*^ mice exhibited RQ > FQ during the dark cycle, relative to controls, whereas only in *Rptor*_*ob*_^*−/−*^ mice was RQ < FQ during the light cycle culminating in a 24-h RQ close to the FQ (Fig. 6d). This suggests that *Rptor*_*ob*_^*−/−*^ mice maintain their constant weight and energy balance through greater substrate flexibility, with greater utilization of fat storage during the light cycle which provide a glycerol substrate for increased gluconeogenesis, thus resulting in higher carbohydrate oxidation in the dark cycle. Conversely, in *Rptor*_*ob*_^*+/−*^ mice, higher carbohydrate oxidation and lipid synthesis in the dark cycle was not offset by greater utilization of fat storage during the light cycle (Fig. 6d). Thus, even with grater substrate flexibility, as is evident in *Rptor*_*ob*_^*−/−*^ mice, higher energy storage during the dark cycle surpassed fat oxidation during the light cycle, resulting in overall continuous weight gain (Fig. 4a, b). In control mice, the average phasic RQ values remained relatively constant (Fig. 6d), which is indicative of fewer shifts in their fat storage and oxidation and a more constant accumulation of adipose tissue mass (Fig. 4a, b).

The increased ability to effectively switch metabolism between carbohydrate and fat oxidation in *Rptor*_*ob*_^*−/−*^ mice was associated with lower circulating TG levels in these mice (Fig. 6e), while FFA levels were higher in *Rptor*_*ob*_^*+/−*^ mice relative to control and *Rptor*_*ob*_^*−/−*^ mice (Supplementary Fig. 7g). Histology revealed less steatosis in the liver of *Rptor*_*ob*_^*−/−*^ mice compared to the controls (Fig. 6h) and significantly lower levels of TG and FFA content (Fig. 6f, g). Furthermore, a significant shift towards adipocyte hypotrophy associated with an upregulation of adipogenic and de novo lipogenic (DNL) genes while no significant changes in the lipolytic genes was observed in both gWAT and iWAT depots from *Rptor*_*ob*_^*−/−*^ mice in response to the HFD (Fig. 6h–l). An increase adipose DNL has been shown to be beneficial for systemic lipid metabolism by sequestering excess TG and thus prevent lipotoxicity in other tissues^30^ suggesting that increased adipose DNL in the *Rptor*_*ob*_^*−/−*^ mice could contribute to the lower levels of circulating and hepatic TG observed in these mice.

Strikingl*y*, we observed an increased multilocularity of lipid droplets, a characteristic of brown adipocytes, in iWAT of *Rptor*_*ob*_^*−/−*^ mice suggesting “browning” of adipocytes (Fig. 6h). Consistent with these changes in morphology, a strong upregulation of *Ucp1* mRNA along with other genes (*Pcg1a*, *Cidea* and *Cox7a*), typically expressed in “browning” WAT, were observed in iWAT of *Rptor*_*ob*_^*−/−*^ mice (Fig. 6m). Immunohistochemistry and Western blot confirmed a significant increase in UCP1 protein levels in *Rptor*_*ob*_^*−/−*^ mice (Fig. 6n, o). We also observed upregulation of *Ucp1* and *Pcg1a* mRNA expression, but not protein, in gWAT of *Rptor*_*ob*_^*−/−*^ mice, whereas no changes in the brown adipose tissue were observed (Supplementary Fig. 8a–d).

### Transcriptional changes associated with deletion of *Rptor* in pre-OBs

**Fig. 7:**
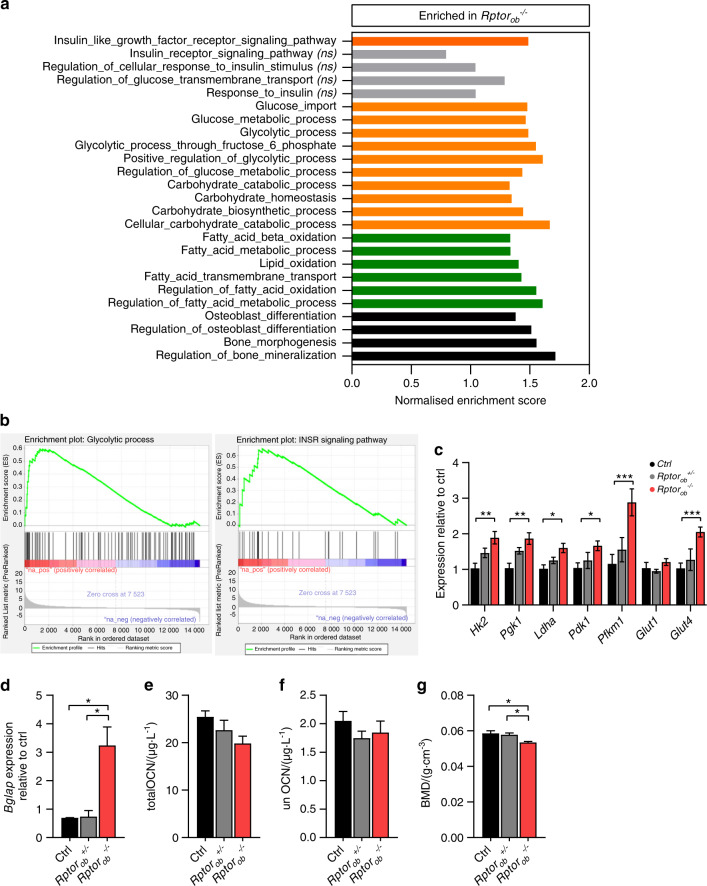
Increased glycolysis and insulin signaling pathways in the bone of HFD-fed *Rptor*_*ob*_^*−/−*^ mice. **a** Gene set enrichment analysis (GSEA) of positively enriched pathways (upregulated) in RNA isolated from combined flushed femur and tibia (*n* = 5; controls versus *Rptor*_*ob*_^*−/−*^). **b** Enrichment plots of glycolysis and insulin-like growth factor receptor signaling pathways. **c** Gene expressions of glycolysis (*HkII*, *Pgk1*, *Ldha*, *Pdk1*, and *Pfkm1*) and glucose transporters (*Glut1* and *Glut4*) in the flushed femur/tibia from HFD-fed mice (*n* = 3–5/genotype). **d** Gene expression of *Bglap* in the flushed femur/tibia, normalized to *Actb* (*n* = 3–4/genotype). **e** Serum levels of total osteocalcin (OCN) and **f** undercarboxylated osteocalcin (unOCN) levels in 18-week-old HFD-fed mice (*n* = 7–15/genotype). **g** Whole-body bone mineral density (BMD) measured by DXA (*n* = 5/genotype). Data are expressed as mean ± SEM. *P < 0.05, **P < 0.01, ***P < 0.001, one-way ANOVA with Tukey’s post hoc test. **a** An orange bar indicates pathways relating to glucose metabolism or insulin signaling, a green bar indicates pathways relating to lipid metabolism, and a black bar indicates pathways relating to bone development. Gray bar (ns) = not significant

RNA sequencing and Gene set enrichment analysis (GSEA) were used to identify transcriptional networks that are differentially regulated in the bones of HFD-*Rptor*_*ob*_^*−/−*^ mice. GSEA of the whole transcriptome revealed positively enriched gene sets for glucose uptake and metabolism and insulin signaling pathways (Fig. 7a, b). After data processing and filtering, 986 genes were found to be differentially expressed with 868 genes significantly upregulated and 118 genes significantly downregulated in the bones of *Rptor*_*ob*_^*−/−*^ mice. These differentially expressed genes (DEGs) were then mapped to specific pathways using KEGG pathway enrichment analysis. Consistent with GSEA, DEGs were highly clustered in glucose metabolism and insulin-responsive pathways including the insulin signaling pathway (15/140 genes; enrichment FDR = 7.95E−04), glycolysis pathway (9/67 genes; enrichment FDR = 1.87E−03) and PI3K-Akt signaling pathway (25/357 genes; enrichment FDR = 8.65E−03). A complete list of upregulated and downregulated gene sets from GSEA and KEGG are detailed in Supplementary Table 1. Gene expression analyses revealed significantly higher levels of genes involved in glycolysis, including *HkII*, *Pgk1*, *Ldha*, *Pdk1*, and *Pfkm1* (Fig. 7c). The expression levels of *Glut4* in the bone of *Rptor*_*ob*_^*−/−*^ mice were twofold higher than the controls, while no differences in *Glut1* expression levels were observed (Fig. 7c). Of note, an increase in glycolytic gene and *Glut4* expression was also observed in NCD-fed *Rptor*_*ob*_^*−/−*^ mice relative to controls (Supplementary Fig. 9a). The higher expression of *Glut4*, together with the upregulation of glycolysis and insulin signaling pathways suggests a potential increase in insulin signaling and insulin-stimulated glucose uptake in the OBs of the *Rptor*_*ob*_^*−/−*^ mice.

### Increased insulin signaling and glycolysis in bone of *Rptor*_*ob*_^*−/−*^ mice

We next interrogated if the systemic changes in glucose metabolism in HFD-fed *Rptor*_*ob*_^*−/−*^ mice was associated with an increase in insulin sensitivity in bone. Gene expression analysis revealed a marked upregulation of *Bglap* in the bone of *Rptor*_*ob*_^*−/−*^ mice (Fig. 7d). However, circulating levels of both total and unOCN were equivalent across all genotypes (Fig. 7e, f). An assessment of bone mass in HFD-fed *Rptor*_*ob*_^*−/−*^ mice revealed a significant reduction (8.7%) in bone mineral density (BMD) as assessed by DEXA (Fig. 7g).

**Fig. 8:**
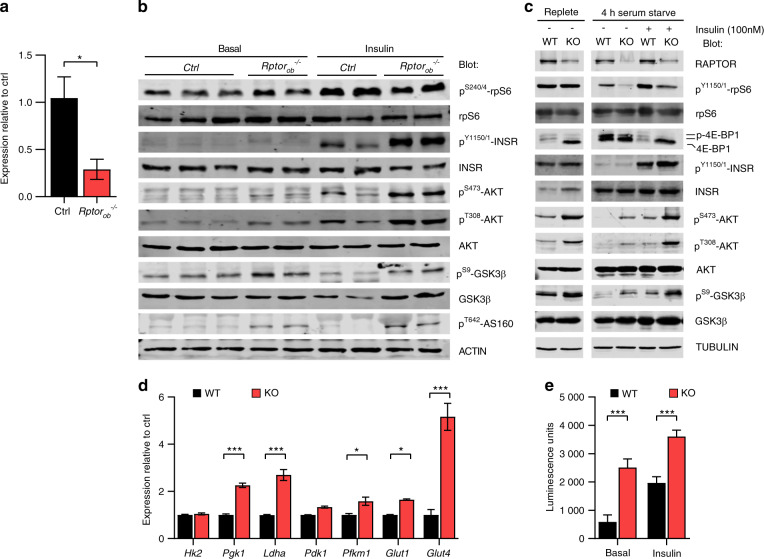
In vivo and in vitro loss of OB-mTORC1 lead to potentiation of OB insulin signaling. **a** Gene expression of *Rptor* in the calvarial bone tissue, normalized to *Actb* (*n* = 3–4/genotype). Data are expressed as mean ± SEM. *P < 0.05, unpaired Student’s *t*-test. **b** Western immunoblot analysis of indicated protein levels in calvarial bone tissue from 18-week-old HFD-fed mice collected after a 6 h fast under either basal (6-h fast + PBS; *n* = 2–3/genotype) or insulin-stimulated (6-h fast + insulin stimulation; *n* = 2/genotype) conditions. **c** Western immunoblot analysis of indicated protein levels in wildtype (WT) and *Rptor* knockout (KO) cultured primary osteoblasts collected under normal growth and a 4-h serum starved conditions before or after stimulation with 100 nmol·L^−1^ insulin. **d** Gene expression analysis of glycolysis and glucose transporters in WT or *Rptor* KO OBs (*n* = 3). **e** 2-Deoxy-D-glucose uptake (measured as units of luminescence) of WT or *Rptor* KO OBs under either basal or insulin-stimulated conditions. Data are expressed as mean ± SD. ***P < 0.001, unpaired Student’s *t*-test

We next directly assessed insulin responsiveness in bone (calvaria) of *Rptor*_*ob*_^*−/−*^ mice. Initially, *Rptor* deletion was confirmed in calvaria by qRT-PCR, using primers specific to floxed exon 6. A significant reduction in *Rptor* gene expression was observed in *Rptor*_*ob*_^*−/−*^ mice consistent with Cre-mediated excision (Fig. 8a). Phosphorylation of INSR at Tyr^1150/1151^ was significantly higher in *Rptor*_*ob*_^*−/−*^ mice (Fig. 8b) whereas phosphorylation of rpS6 at Ser^240/244^ was blunted consistent with reduced mTORC1 activity. Importantly, both basal and insulin-stimulated phosphorylation levels of AKT (Thr^308^ and Ser^473^) were significantly increased in *Rptor*_*ob*_^*−/−*^ mice (Fig. 8b). This was associated with increased phosphorylation of AKT substrates, glycogen synthase kinase (GSK)-3β Ser,^9^ and AS160 Thr^642^ (which regulates insulin-stimulated GLUT4 trafficking) (Fig. 8b). This data, in combination with the RNA-seq data, suggests that loss of mTORC1 function in OBs increases basal and insulin-dependent glucose uptake in *Rptor*_*ob*_^*−/−*^ mice.

To address this question, we generated *Rptor* knockout OBs (*Rptor*^*KO*^) in vitro.^14^ Deletion of *Rptor* caused a reduction in basal and insulin-stimulated phosphorylation of the key mTORC1 substrates, p70S6K and 4E-BP1 and rpS6, findings consistent with reduced mTORC1 activity (Fig. 8c). Deletion of *Rptor* was also associated with an increase in basal and insulin-stimulated AKT activation and phosphorylation of GSK-3β at Ser,^9^ (Fig. 8c), consistent with the in vivo insulin stimulation experiments (Fig. 8b). Furthermore, qRT-PCR analysis revealed an increase in the expression of glycolytic genes in *Rptor*^*KO*^ cells relative to wildtype control cells mirroring in vivo findings (Fig. 8d). Finally, using the accumulation of 2-deoxyglucose as a surrogate measure of glucose uptake, loss of mTORC1 function in OBs increased both basal and insulin-stimulated glucose uptake into OBs (Fig. 8e). Taken together, our findings support a central role for the mTORC1 complex in OBs in the control of systemic glucose metabolism and highlight the importance of this complex in bone in the development of systemic insulin resistance.

## Discussion

**Fig. 9:**
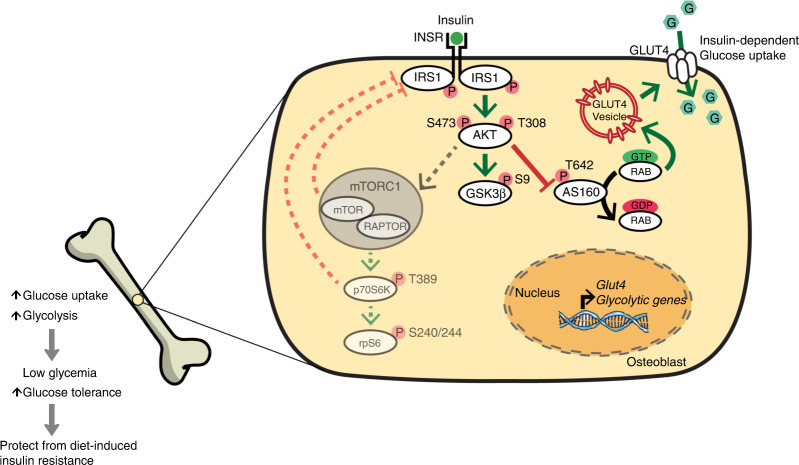
Schematic: Suppression of mTORC1 activity in pre-OBs results in relaxation of mTORC1-dependent negative feedback control (both directly and indirectly via activation of p70S6K) of insulin signaling by disrupting the interaction between IRS1 and INSR. This lead to an enhanced and prolonged insulin signaling as indicated by hyperactivation of AKT at Ser^473^ and Thr^308^ in both basal and insulin-stimulated states. AKT hyperactivation attenuates the inhibitory effects of the AS160 on RAB leading to GLUT4 membrane translocation. These molecular changes are associated with an increased cellular glucose uptake in the *Rptor* KO OBs and upregulation of an insulin-dependent glucose transporter 4 (*Glut4*) and glycolysis-promoting enzymes in the skeleton of *Rptor*_*ob*_^*−/−*^ mice. Systemically, *Rptor*_*ob*_^*−/−*^ mice showed persistently low fasting glucose levels and significantly increased tolerance to glucose; the metabolic phenotypes which are, at least in part, attributable to an enhanced responsiveness to insulin and insulin-dependent glucose uptake in OBs

In this study, we show that NCD-fed *Rptor*_*ob*_^*−/−*^ mice displayed a marked metabolic phenotype characterized by low fat mass, hypoglycemia, enhanced glucose tolerance, and increased insulin sensitivity. These beneficial metabolic effects were maintained under excess nutrient conditions resulting in protection against HFD-induced obesity and insulin resistance. Furthermore, we provide evidence that these phenotypes are, at least in part, attributable to an enhanced responsiveness to insulin and insulin-dependent glucose uptake in OBs (Fig. 9).

### Skeletal mTORC1, insulin signaling, and glucose uptake

Previous studies have shown that modulation of insulin signaling in OBs, by genetically decreasing or increasing the levels of INSR expression, either worsens or enhances glucose tolerance and insulin resistance in HFD-fed animals, respectively.^31^ Furthermore, mice with OB-specific deletion of *FoxO1 (FoxO1*^OB*−/−*^), a negative regulator of insulin signaling, are protected from HFD-induced obesity.^3^ Consistent with these data, suppression of skeletal mTORC1 activity, a downstream mediator and negative regulator of insulin signaling, protects mice from diet-induced obesity. Of note, the lower fasting glucose levels, enhanced glucose tolerance and increased insulin sensitivity displayed by *Rptor*_*ob*_^*−/−*^ mice mimics the metabolic phenotype of mice that overexpress the human INSR in the OBs (*Col1a1-INSR*^*Tg*^ mice).^31^ At the molecular level, we did not observe enhanced insulin-stimulated activation of AKT, an important effector of insulin signaling, in any peripheral insulin-targeted tissues, however, insulin signaling in the bone of *Rptor*_*ob*_^*−/−*^ mice was significantly enhanced. Similarly, improved glucose tolerance and insulin sensitivity in *Col1a1-INSR*^*Tg*^ mice were only associated with enhanced insulin signaling in bone.^31^

Several lines of evidence point to the potentiation of insulin signaling as being an important mechanism in the metabolic phenotype of *Rptor*_*ob*_^*−/−*^ mice. Firstly, using bones isolated from HFD-fed mice, RNA-seq and GSEA revealed an upregulation of genes involving in glycolysis pathway. This gene expression pattern was also observed in the bones of NCD-fed *Rptor*_*ob*_^*−/−*^ mice and *Rptor* KO calvarial cells in vitro. Secondly, basal and insulin-stimulated levels of AKT and AS160 were evident in calvarial cells of HFD-fed *Rptor*_*ob*_^*−/−*^ mice in vivo. Hyperphosphorylation of AKT at Ser^473^ and Thr^308^ was also observed in vitro in *Rptor* KO OBs, in both basal and insulin-stimulated states. Thirdly, glucose uptake was significantly increased in *Rptor* KO cells in vitro in both basal and insulin-stimulated states, suggesting that constitutive activation of AKT leads to an increase in glucose transport in the absence of mTORC1 activity. Several studies have now recognized bone as a significant site of glucose uptake accounting for up to 15% of steady state levels and the magnitude of glucose uptake by OBs is sufficient to impact systemic glucose disposal in mice.^19,32,33^ The higher basal phosphorylation levels of AKT and AS160 in vivo suggests that an increase in basal skeletal uptake of glucose could account for the low glycemic phenotype of *Rptor*_*ob*_^*−/−*^ mice (Fig. 9).

### Metabolic phenotypes independent of OCN

The improved metabolic phenotype of NCD-fed *Rptor*_*ob*_^*−/−*^ mice appears to contradict the widely held view that low unOCN levels are associated with poor metabolic outcomes. Circulating OCN levels are inversely correlated with plasma glucose levels, fat mass and the extent of metabolic impairment in both animal and human studies.^6,34^ Furthermore, *Ocn*-deficient mice (*Ocn*^*−/−*^) exhibit higher adiposity, hyperglycemia, hypoinsulinemia, impaired insulin secretion and sensitivity and are glucose intolerant,^6^ a metabolic phenotype opposite to that observed in *Rptor*_*ob*_^*−/−*^ mice. The defects in glucose homeostasis in *Ocn*^*−/−*^ mice are accompanied by impaired pancreatic β-cell proliferation and insulin secretion and the development of insulin resistance in other insulin-target tissues.^6^ While the hypoinsulinemia observed in our *Rptor*_*ob*_^*−/−*^ mice may be attributed to low serum levels of unOCN, GSIS tests indicated maintenance of ability to secrete insulin in response to glucose in the *Rptor*_*ob*_^*−/−*^ mice. The low serum levels of OCN observed in NCD-fed *Rptor*_*ob*_^*−/−*^ mice are likely secondary to the stall in osteogenesis that occurs in response to reduced mTORC1 activity in pre-OBs.^14,35^ While we cannot rule out the possibility that the metabolic improvements observed in *Rptor*_*ob*_^*−/−*^ mice are due to low levels of OCN priming its target tissues to become highly sensitive to OCN, this is unlikely to be the case, as serum unOCN levels were reduced in both *Rptor*_*ob*_^*+/−*^ and *Rptor*_*ob*_^*−/−*^ mice, but only *Rptor*_*ob*_^*−/−*^ mice displayed an improved metabolic phenotype. Furthermore, in contrast to other animal models (i.e., *FoxO1*^OB*−/−*^ and *Col1a1-INSR*^*Tg*^ mice) where protection from HFD-induced glucose intolerance and insulin resistance were attributed to an insulin-mediated increase in bone resorption leading to an increase in OCN activity and serum levels (although no difference in *Ocn* mRNA expression was observed),^31^ both RNA-seq and qRT-PCR analysis of bone samples from HFD-fed *Rptor*_*ob*_^*−/−*^ mice revealed a downregulation of genes involved in bone resorption and osteoclast differentiation pathways, and upregulation of genes associated with bone formation and OB differentiation. Intriguingly, despite detection of significantly higher *Bglap* mRNA expression levels in HFD-fed *Rptor*_*ob*_^*−/−*^ mice, circulating levels of both total and unOCN protein were equivalent to those of the controls. At this stage, the mechanisms underlying this disparity remain to be determined. OCN is expressed exclusively from mature OBs and low serum levels are used as a marker of reduced OB activity and bone mass.^36^ Low serum OCN levels correlated with the low bone mass phenotype of normal chow-fed *Rptor*_*ob*_^*−/−*^ mice,^14^ while no difference in BMD was observed in HFD-fed *Rptor*_*ob*_^*−/−*^ mice relative to controls (Fig. 7g). Previous studies have shown that HFD-fed wild-type mice have reduced total and unOCN levels,^31,37^ suggesting that the apparent “normalization” of OCN levels in HFD-fed *Rptor*_*ob*_^*−/−*^ mice may simply reflect this reduction. Another possibility is that OB-mTORC1 is a negative regulator of *Bglap* gene expression and possibly required for *Bglap* translation. Notwithstanding this, an important finding of this present study is the observation that the beneficial metabolic effects associated with OB-mTORC1 inhibition occur independently of OCN. Several studies have implicated OCN-independent mechanisms in the bone-metabolism interplay. For example, genetic ablation of OBs, or conditional OB-specific inactivation of GSK-3β, β-catenin or *Vhl*, a regulator of Hypoxia-inducible factors (HIFs), leads to systemic metabolic alterations that cannot be fully rescued or explained by congruent changes in serum OCN.^19,38–40^ These studies suggest the existence of additional, as yet unidentified, bone secretagogues, amongst other factors, that influence global glucose homeostasis and energy metabolism.

### Energy metabolism and whole-body metabolic flexibility

In NCD-fed *Rptor*_*ob*_^*−/−*^ mice, the failure to build up their energy store (as evident by a marked reduction in body fat and accumulation of small adipocytes in the fat depots) is likely due to their increased energy expenditure, with unaltered caloric intake, and the preference for fat oxidation (as indicated by the relatively lower RQ). This lipoatrophy could represent an adaptive response to the concurrent low insulin and glucose levels which, together, increase the production of ketone bodies,^41^ as an alternative energy source, by increased mobilization of fatty acid from the fat storage to the liver, in order to prevent hypoglycemic death. In support of this, recent studies have shown that during short-term starvation in lean rats, both hypoleptinemia and insulinopenia are necessary to maintain euglycemia and thus promote survival.^42^ Hypoleptinemia stimulates secretion of corticosterone which, in the presence of hypoinsulinemia, leads to WAT lipolysis and the shift from whole-body carbohydrate to fat/ketone oxidation.^42^ Furthermore, in NCD-fed *Rptor*_*ob*_^*−/−*^ mice, the observed changes in body composition (i.e., a significant reduction in total body weight and fat mass) while paradoxically increasing BMAT are commonly observed under states of starvation including dietary restricted mouse models^43^ and in human anorexia nervosa patients.^44^ A growing number of studies now attribute the beneficial effects of BMAT expansion to adiponectin production under restricted caloric conditions^22,43,45^ and, as such, elevated serum adiponectin levels could contribute to the improved metabolic phenotype of *Rptor*_*ob*_^*−/−*^ mice. Adiponectin has been reported to inhibit gluconeogenesis and increase fatty acid beta-oxidation in the liver to facilitate its insulin-sensitizing effects.^46,47^ Of note, the upregulation of genes involved in fatty acid beta-oxidation (*Cpt1a*, *Acox*, and *Acc2*) was observed in the liver of *Rptor*_*ob*_^*−/−*^ mice (Supplementary Fig. 3a). The lack of differences in the *Pepck* and *G6p* expression levels in the *Rptor*_*ob*_^*−/−*^ mice may be attributable to their persistently high circulating adiponectin levels (both in the fed (Fig. 3e) and fasting states (data not shown)). Furthermore, the collection of tissues during fasting could potentially mask any potential differences as fasting would lead to the upregulation of *Pepck* in the control mice.^48^ Adiponectin has been reported to increase fatty acid beta-oxidation in liver to facilitate its insulin-sensitizing effects.^46,47^ These increases in fatty acid oxidation genes in the liver were indeed consistent with the lower respiratory quotient (Fig. 1m, n) observed in these mice.

*Rptor*_*ob*_^*−/−*^ mice are resistant to diet-induced weight gain and maintained a constant body weight, after 6 weeks of HFD, despite consuming a similar amount of food to the controls. Importantly, the reduced adiposity (Fig. 4c, d) and lack of adipocyte hypertrophy (Fig. 6h–j) in the *Rptor*_*ob*_^*−/−*^ mice were observed despite an upregulation of lipogenic genes in these tissues. Downregulation of lipogenic genes upon HFD feeding or in obesity is associated with the development of adipocyte insulin resistance^49–52^ and thus the upregulation of these genes in the *Rptor*_*ob*_^*−/−*^ mice is likely a secondary effect of their ability to retain sensitivity to insulin. Both lipogenesis and adipogenesis are well-known insulin-regulated processes in adipose tissues^53^ and consistent with this, a significant upregulation of *Pparg*, an important regulator of adipogenesis, was also observed in the adipose tissue of *Rptor*_*ob*_^*−/−*^ mice (Fig. 6k, l). No significant changes in the expression levels of *Atgl*, an important lipolytic gene, was observed the adipose tissue of *Rptor*_*ob*_^*−/−*^ mice which is consistent with a higher lipogenesis-to-lipolysis ratio as previously reported in smaller insulin-sensitive adipocytes.^51^ Finally, an increased sensitivity to insulin in adipose tissue of *Rptor*_*ob*_^*−/−*^ mice is evidenced by a significant, or a trend toward significant, increase in insulin-stimulated rpS6 phosphorylation (Fig. 5c, d, g, h) and the upregulation of *Glut4* transcripts (Supplementary Fig. 10).

The mechanism(s) underlying the browning of iWAT in *Rptor*_*ob*_^*−/−*^ mice appears to be independent of previously reported browning agents including fibroblast growth factor 21 (FGF21)^54^ and muscle-secreted irisin^55^ as we observed no changes in the expression levels of these factors (data not shown). It is interesting to note that we did observe an upregulation of two bone-secreted factors, bone morphogenetic protein 7 and Wnt-signaling inhibitor, sclerostin (Supplementary Fig. 9b, c), both of which have been implicated in the browning of WAT.^56,57^ With regards to sclerostin, mTORC1 has been shown to negatively regulate SOST in osteocytes via activation of SIRT1, a negative regulator of *Sost* gene expression^58^ linking mTORC1 function in OBs/osteocytes with maintaining systemic energy balance.

We propose that the protection from diet-induced weight gain is partially attributable to their ability to switch substrate oxidation, while maintaining energy balance. *Rptor*_*ob*_^*−/−*^ mice exhibited a greater capacity to switch between oxidizing fatty acids and carbohydrates during the light and dark cycles, under ad libitum feeding, suggesting they are capable of maintaining metabolic flexibility (defined as the increase in RQ between fasting and postprandial states). In humans, impairments in metabolic flexibility have been associated with obesity, the development of insulin resistance and diabetes (reviewed in ref. ^59^). Furthermore, clinical studies report lower fat oxidation and higher RQ in subjects with a family history of diabetes,^60^ and, in particular, a smaller decrease in RQ was observed during the sleeping period in these subjects.^61^ These results, combined with the impaired glucose metabolism observed in the control mice, indicate a correlation between metabolic inflexibility and abnormality in glucose homeostasis. As such, the flexibility in substrate switching in *Rptor*_*ob*_^*−/−*^ mice could produce the systemic metabolic protection phenotype observed in these mice.

### Inhibition of mTORC1 as a novel therapeutic approach to treat T2DM

To our knowledge, this is the first report to describe the metabolic phenotype associated with loss of mTORC1 function in OBs. In other insulin-responsive tissues, loss of mTORC1 function has positive and negative effects on whole-body metabolism. In WAT, deletion of *Rptor* using *Ap2-Cre* (*raptor*^*ad−/−*^) resulted in lean mice that were resistant to the negative effects of HFD-induced obesity on glycemic control and HFD-induced hyperphosphorylation of S6K1.^12^ In contrast, deletion of *Rptor* in mature adipocytes (*AdipoQ-Cre*) led to lipodystrophy and insulin resistance that was associated with a significant increase in liver mass and ectopic lipid accumulation.^13^ Inactivation of *Rptor* in muscle (RAmKO mice) results in protection from HFD-induced weight gain and enhanced glucose tolerance without improvement in insulin sensitivity.^62^ Reduced S6K1 activation and increased phosphorylation of AKT and AS160 were also observed in the muscle of RAmKO mice. However, this was associated with a significant decrease in the expression of glucose transporters and glycolytic genes and an increase in glycogen synthesis and muscle glycogen levels.^11,62^ Conversely, liver-specific deletion of *Rptor* (*Raptor*^*Δhep*^ mice) resulted in severe liver damage with augmented inflammation and fibrosis while glucose tolerance was improved only in NCD-fed mice.^63^ These tissue-specific functions and responses to mTORC1 inhibition are further highlighted in studies where administration of rapamycin has been shown to have both beneficial^64,65^ and detrimental effects on metabolism^66–69^ demonstrating that sustained systemic suppression of mTORC1 function has significant limitations.

Skeletal involvement in T2DM is well established with T2DM patients displaying an increased risk of fracture despite higher femoral neck and lumbar spine BMD.^70,71^ At the microarchitecture level, an increase in cortical porosity and bone micro-fractures has been observed in T2DM patients which may reflect disrupted bone remodeling.^72^ Indeed, a decrease in the number and differentiation of OBs, leading to diminished quantities of osteoid is considered an important driver of skeletal change in T2DM.^73^ While a role for OB-mTORC1 in these changes requires further investigation, hyperactivation of mTORC1 via deletion of *Tsc2* in OBs (∆Tsc2) results in mice with a high bone mass phenotype and insulin resistance.^17^ These phenotypes are associated with an accumulation of poorly differentiated OBs and disorganized bone structure. Similarly, an accumulation of poorly organized sclerotic bone occurs in patients with tuberous sclerosis, an autosomal dominant disorder caused by the mutation of TSC1 or TSC2 leading to hyperactivation of mTORC1.^74^ While further evaluation of the role of mTORC1 in OBs in the development of diet-induced insulin resistance is required, the protective phenotypes observed in HFD-fed mice with either pre- or post-natal deletion of *Rptor* in OBs suggest OB-mTORC1 is a potential target for the development of antidiabetic drugs, whereby localized inhibition of skeletal mTORC1 could have favorable metabolic outcomes in patients with T2DM.

In summary, our studies have demonstrated that suppression of skeletal mTORC1 signaling in mice leads to a dramatic improvement in glucose metabolism and protection from diet-induced obesity and insulin resistance. Collectively, these results point to a critical role for the mTORC1 complex in OBs in integrating whole-body nutrient status and local insulin signaling in order to maintain systemic glucose homeostasis.

## Materials and methods

### Transgenic mice and diet

All mice were bred and group-housed (maximum five mice/cage) in pathogen-free conditions at the SAHMRI Bioresources Facility (Adelaide, Australia) under a 12 h light–dark cycle (lights on at 06:00) and constant temperature (20–23 °C) and ad libitum access to a standard chow diet (Teklad Global Diet #2918: 18.6% protein, 6.2% fat; Harlan, IN, USA) and water. For diet-induced obesity studies, mice were fed a HFD (Specialty Feeds #SF16-096: 19.4% protein, 23% fat (43.4% kcal from fat); WA, Australia) from weaning to 18 weeks of age. For postnatal deletion studies, pregnant dams and pups were maintained on doxycycline chow (Specialty Feeds #SF08-026: 600 mg Doxycycline per kg; WA, Australia). At 4 weeks of age, pups were placed on a HFD (Specialty Feeds #SF16-096; WA, Australia) until 18 weeks of age. FQ was estimated by linear regression analysis of changes in body mass (*∆M*: *x*-axis) and 24-h RQ (*y*-axis); the y-intercepts were the 24-h RQ when *∆M* equaled zero, or theoretical FQ^29,75^ (Supplementary Fig. 5). All studies were performed with Institutional Ethics approval (SAHMRI Animal Ethics Committee, #SAM164). Male conditional knockout mice in which *Rptor* was disrupted in early osteoprogenitor cells were generated using Osx1-GFP::Cre mice,^24^ R26eYFP mice,^76^ and *Rptor*^*fl/fl*^ mice^11^ as previously described.^14^ Animals were weighed twice weekly for the duration of the study and at the end of the study, body length and whole-body lean and fat mass were measured postmortem using a dedicated mouse DXA (Lunar Piximus II, GE Medical Systems, Madison WI, USA).

### Metabolic phenotyping

ITTs and glucose tolerance tests (GTTs) were performed following intraperitoneal injection of 0.75 U·kg^−1^ insulin (Novo Nordisk Pharmaceuticals, Australia) or 2 g·kg^−1^ glucose, respectively after a 6-h fast (07:00–13:00). Blood glucose levels were measured at indicated time points using a handheld glucometer (Accu-Chek, Roche, Australia). At 0- and 30-min time points during the GTT, ~50 µL of whole blood was collected, serum isolated and then immediately frozen at −80 °C for later assessment of glucose-stimulated insulin levels by ELISA (EZRMI-13K, Millipore, MA, USA) as per manufacturer’s instructions.

### Indirect calorimetry and body composition analyses

Indirect calorimetry assessments were performed using the Promethion metabolic cage system (Sable Systems, NV, USA). Mice were single-housed in Promethion cages for 72–96 h with ad libitum access to food and water. Monitoring was performed for 48 h following an initial 24-h acclimatization period. RQ was calculated as the ratio of VCO_2_/VO_2_.

### Fecal lipid assessment

Dried feces (1 g) were collected from single-housed mice over a 72-h period, pulverized using a mortar and pestle and rehydrated in 5 ml of normal saline. A 2:1 chloroform: methanol solution was used to extract lipids from the suspension as previously described.^77^ After lipid extraction and a 4-d evaporation of the organic phase, the remaining lipid mass was weighed using analytical balance. The lipid content was calculated as the percentage of the total fecal weight.

### ELISA

Non-fasted (for chow studies) and fasted (for HFD studies) blood samples were collected (12:00–14:00) via cardiac puncture into Minicollect tubes (Greiner Bio One, Kremsmünster, Austria), centrifuged at 845 × *g* for 10 min at room temperature and extracted serum stored at −80 °C. Commercial ELISA kits were used for the measurement of: TGs and FFA (ab65336 and ab65341, respectively, Abcam, MA, USA), leptin and adiponectin (EZML-82K and EZMADP-60K, respectively, Millipore, MA, USA), OCN (BT-470, Alfa Aesar, Lancashire, UK), unOCN (MK129, Takara, Japan), and LCN2 (RDSMLCN20, R&D Systems, Minneapolis, MN, USA) as per manufacturer’s instructions.

### Histology and Immunohistochemistry

Tissues were dissected, fixed in 10% formalin and embedded in paraffin. Hematoxylin and eosin staining was performed on 5 μm adipose tissue sections. Image J was used to measure adipocyte cell size and distribution and was presented as a percentage of total cells from *n* = 5 images per mouse. Immunohistochemistry was performed on iBAT using rabbit anti-UCP1 (ab209483, Abcam, Cambridge, MA, USA, 1:5 000). For pancreatic tissues, immunohistochemistry was performed using rabbit anti-insulin (ab209483, Abcam, 1:1 000). Total pancreatic surface and β-cell area (area positive for insulin immunostaining) were quantified from at least five sections per pancreas (each 50 µm apart) using a DP72 camera (Olympus, Tokyo, Japan) attached to a Leica microscope (Wetzlar, Germany) and Osteomeasure software (Osteometrics, GA, USA). For Oil red O staining, liver samples were mounted in Tissue-Tek Optimum Cutting Temperature compound (Sakura Finetek, The Netherlands). Cryosections were cut at 10 μm and Oil red O staining was performed as previously described.^78^

### Protein isolation and western blotting

For insulin signaling studies, mice were fasted for 6 h (07:00–13:00) prior to administration of insulin (150 mU·g^−1^ BW, i.p) or PBS control. After 20 min, mice were euthanized and tissues were harvested, snap-frozen in liquid nitrogen and stored at −80 °C. Tissue samples were lysed in modified RIPA buffer with addition of protease inhibitors (cOmplete™, Roche, Basel, Switzerland) and homogenized using a TissueRuptor (QIAGEN, Victoria, Australia). Equal amounts of protein (50 µg,) were resolved by SDS-PAGE and transferred to Immobilon-FL PVDF Membrane (MerckMillipore, Darmstadt, Germany). Antibodies for immunoblotting are listed in Supplementary Table 2. After incubation with fluorescently tagged secondary antibody, membranes were scanned using a Li-Cor Odyssey imaging system (LI-COR Biosciences, NE, USA). Quantitative analysis was performed using Image Studio software LI-COR Biosciences. Protein levels were quantified and levels normalized to a loading control. The analysis of adiponectin multimers in serum was performed as previously described.^79^

Full images of western blots are included in Supplementary Fig. 11.

### RNA isolation and quantitative RT-PCR

All RNA extractions were carried out using TRIzol reagent (Sigma) according to manufacturer’s instructions. Total RNA (1.5 µg) was reverse transcribed into cDNA using Superscript IV Reverse Transcriptase (Invitrogen, CA, USA). Real-time PCR reactions were performed using RT^2^ SYBR Green ROX reagent (QIAGEN, Hilden, Germany) in a CFX Connect™ Real-Time PCR machine (Bio-Rad). Forward and reverse primer pairs are listed in Supplementary Table 3. Relative mRNA expression was determined using the 2^–∆∆Ct^ method.^80^

### RNA high-throughput sequencing and gene expression analysis

RNA isolated from marrow-flushed combined femur/tibia were processed and sequenced through the low-input RNA-seq pipeline at The David Gunn Genomics Facility, SAHMRI (Illumina Nextseq, San Diego, CA, USA) with a 75 cycle v2 High Output sequencing kit. Analyses were then conducted by SAHMRI Bioinformatics Facility. Initial raw read processing was performed using an in-house pipeline developed at SAHMRI. Raw 75 bp single-end FASTQ reads were assessed for quality using FastQC and results aggregated using R/Bioconductor package ngsReports.^81^ Reads were then trimmed for sequence adapters using AdapterRemoval^82^ and aligned to the GRCh38/mm10 version of the mouse genome using the RNA-seq alignment algorithm STAR.^83^ After alignment, mapped sequence reads were summarized to the mm10 gene intervals using featureCounts,^84^ and count table transferred to the R statistical programming environment for expression analysis.

Gene counts were filtered for low expression counts by removing genes with less than 1 count per million in more than four samples and then normalized by the method of trimmed mean of M-values.^85^ Differential gene expression was carried out on log-CPM counts and precision weights available from the limma voom function^86^ and edgeR.^87,88^ GSEA was performed from Molecular Signature Database and other gene sets available from the limma package.^89^ Gene sets returning an FDR adjusted *P* value < 25% were accepted as statistically enriched.

### Primary cell culture and insulin pulse

Primary calvarial OB cultures were established from new born (P4) *Rptor*^*fl/fl*^ mice and *Rptor* knockout cells generated as previously described.^14^ For insulin pulse experiments, cells were incubated under serum-free conditions for 4 h then pulsed with 100 nmol·L^−1^ insulin (ProSpec-Tany TechnoGene Ltd, Ness-Ziona, Israel) for 10 min. RNA and protein was extracted from wild-type and *Rptor* KO cells, under normal growth and serum starved conditions before or after stimulation with 100 nmol·L^−1^ insulin, as described above. qRT-PCR and western blotting were performed as described above.

### Glucose uptake assays

Basal and insulin-stimulated glucose uptake was performed using the Glucose Uptake-Glo^TM^ assay (Promega, Madison, WI, USA). Briefly, wildtype and *Rptor* knockout cells (1 × 10^4^/well) in clear bottom 96 wells plates (Corning, New York, USA) were allowed to adhere overnight then serum deprived for 4 h. Cells were then stimulated with 100 nmol·L^−1^ insulin in glucose- and serum-free DMEM (ThermoFisher) supplemented with 10 nmol·L^−1^ Hepes, pH 7.4, and 1 nmol·L^−1^ sodium pyruvate for 1 h at 37 °C/5% CO_2_. Media was replaced with 50 µL/well of 2-deoxyglucose (1 nmol·L^−1^) for 10 min at 25 °C. Subsequent steps were performed according to the manufacturer’s instructions. Luciferase activity was detected using a GloMax^®^ Luminometer (Promega).

### Statistical analysis

All data are presented as mean ± standard error of the mean. Statistical analyses were performed using a one-way or two-way ANOVA with a Tukey’s post hoc test or an unpaired Students’s *t*-test using GraphPad Prism (GraphPad Software Inc, CA, USA). Significance was accepted at *P* < 0.05, with asterisks denoting *P* value levels: *P < 0.05; **P < 0.01; ***P < 0.001.

## Supplementary information

Supplementary Figure 1

Supplementary Figure 2

Supplementary Figure 3

Supplementary Figure 4

Supplementary Figure 5

Supplementary Figure 6

Supplementary Figure 7

Supplementary Figure 8

Supplementary Figure 9

Supplementary Figure 11

Supplementary Figure 10

Supplementary Table 1_final.xlsx

Supplementary Table 2_final.docx

Supplementary Table 3_final.docx
