# Supplementary figures and images for: The mTORC1 complex in pre-osteoblasts regulates whole-body energy metabolism independently of osteocalcin
Source: Bone Res. 2021 Feb 8;9:10. doi: 10.1038/s41413-020-00123-z (PMC7868369; doi:10.1038/s41413-020-00123-z)

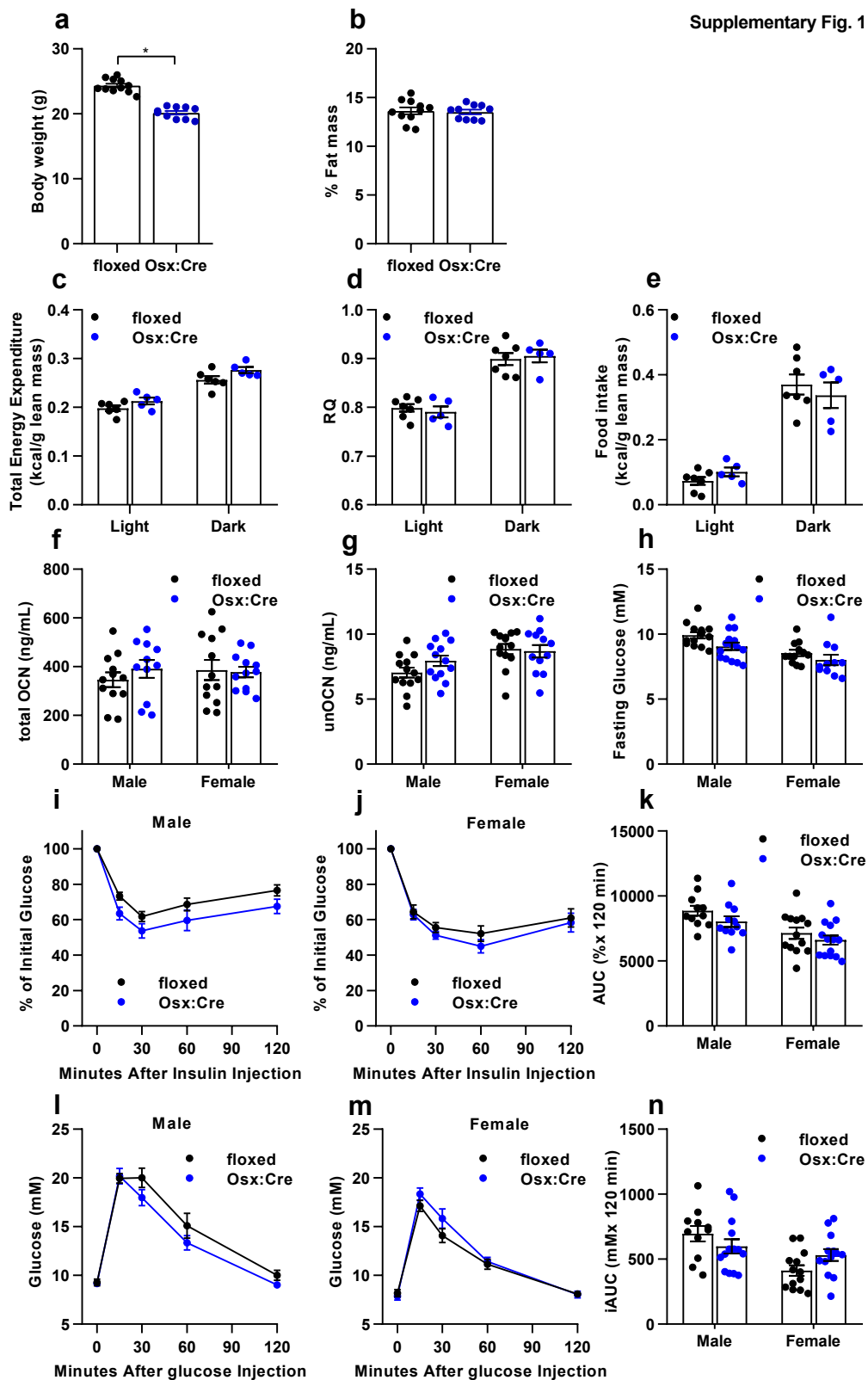

Supplement: Supplementary file 1 — Supplementary Figure 1 [file 41413_2020_123_MOESM1_ESM.pdf]

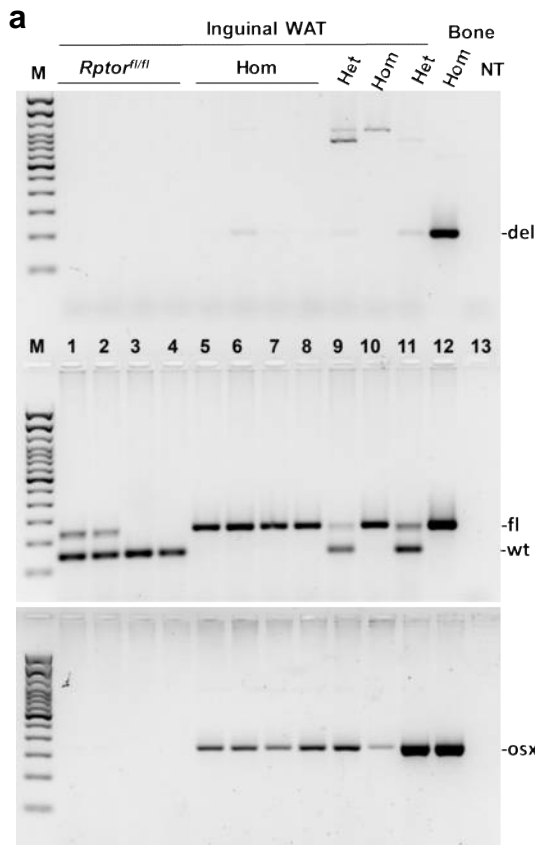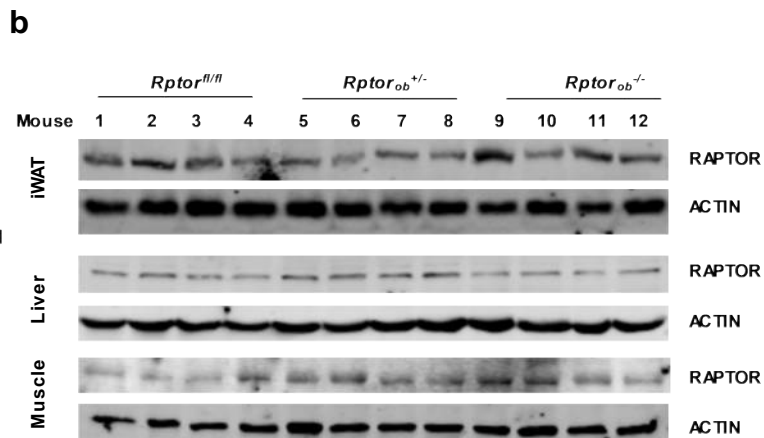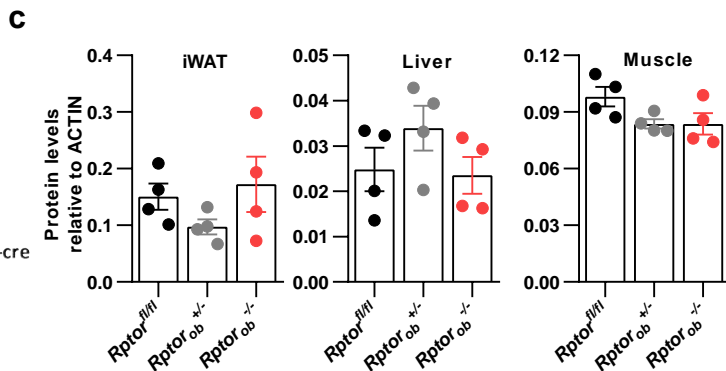

Supplement: Supplementary file 2 — Supplementary Figure 2 [file 41413_2020_123_MOESM2_ESM.pdf]

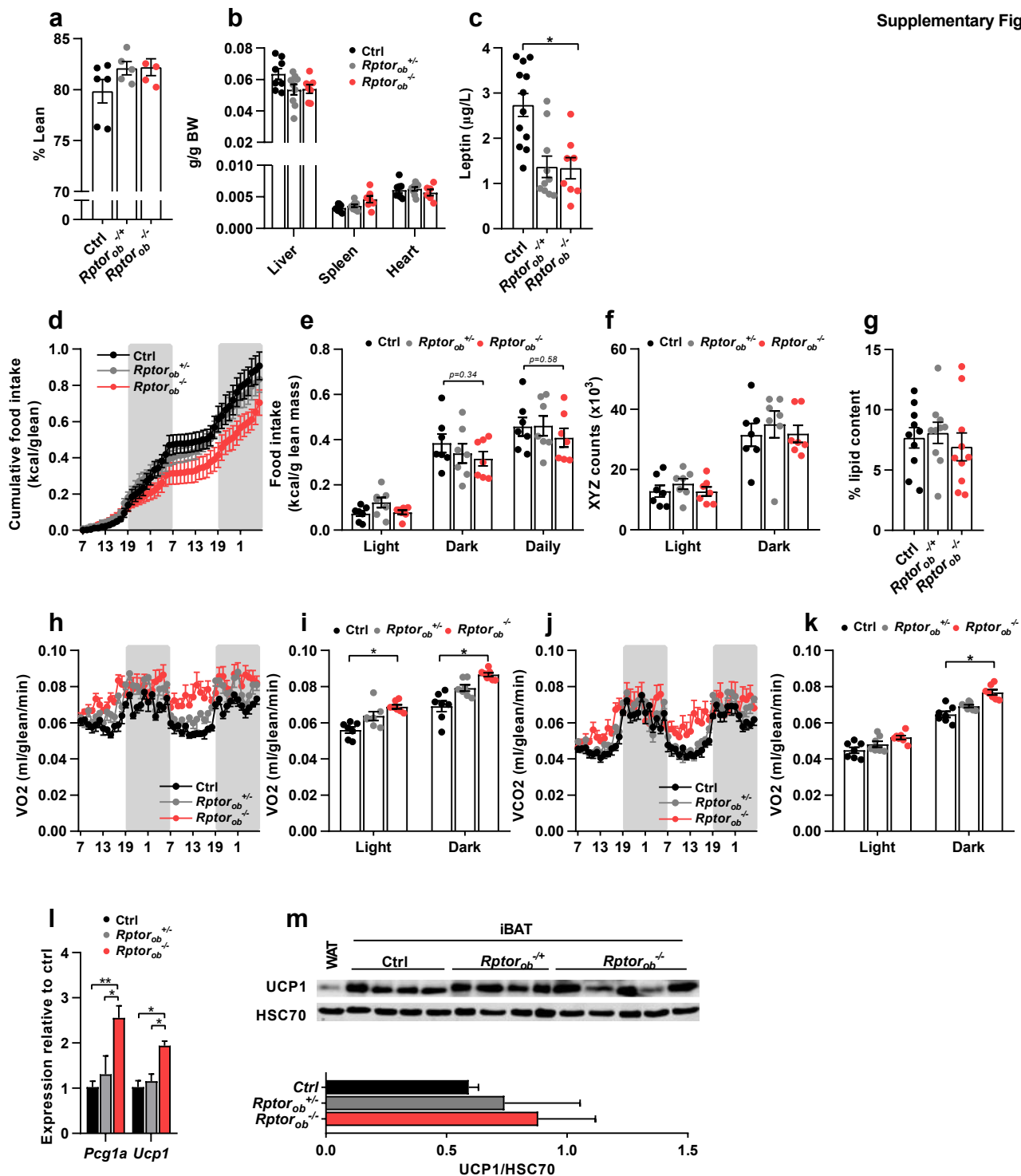

Supplement: Supplementary file 3 — Supplementary Figure 3 [file 41413_2020_123_MOESM3_ESM.pdf]

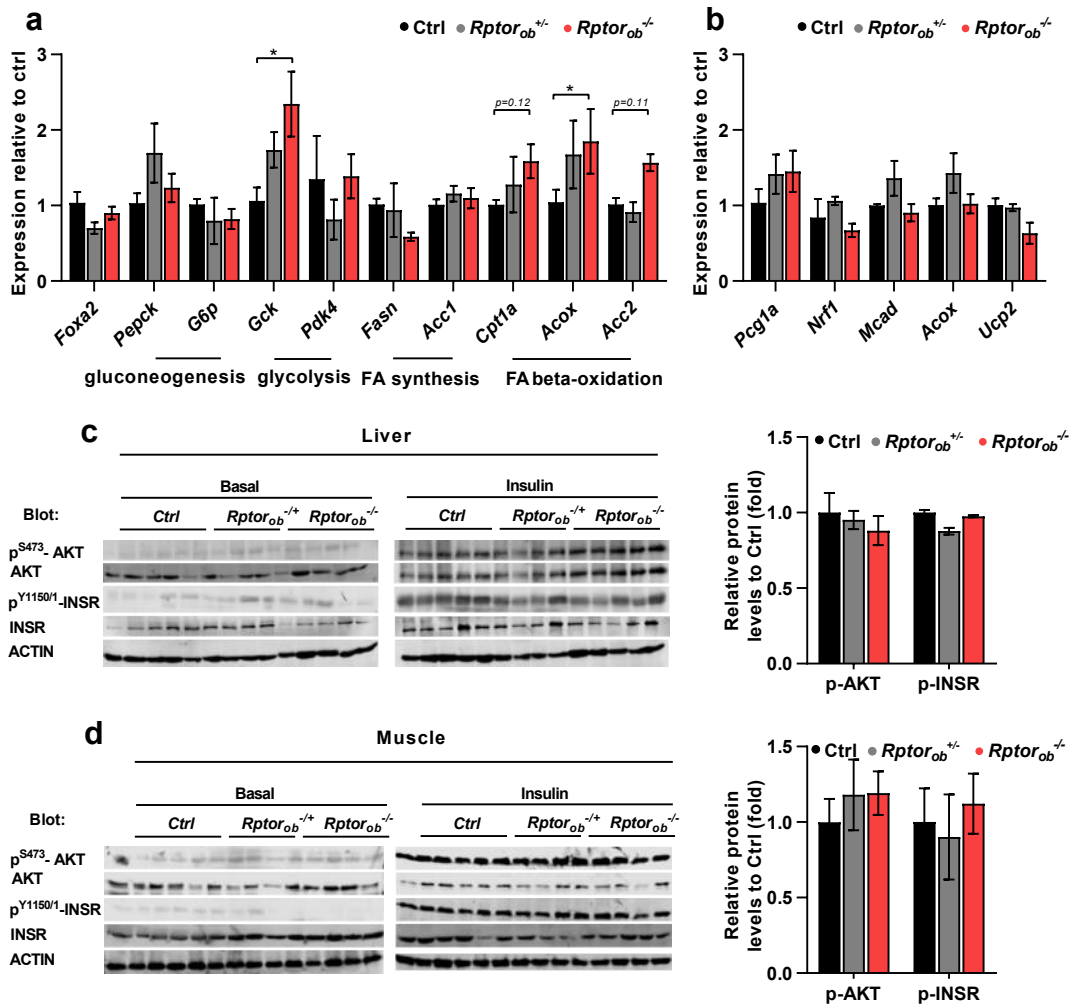

Supplement: Supplementary file 4 — Supplementary Figure 4 [file 41413_2020_123_MOESM4_ESM.pdf]

Supplementary Fig. 5

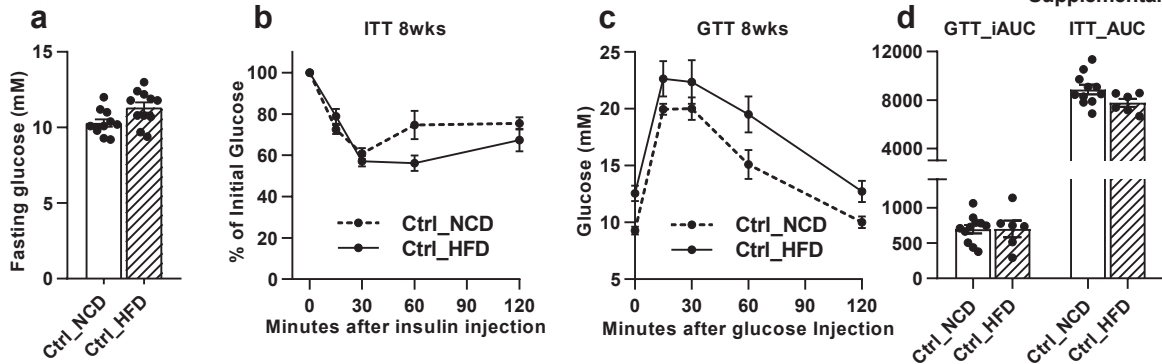

Supplement: Supplementary file 5 — Supplementary Figure 5 [file 41413_2020_123_MOESM5_ESM.pdf]

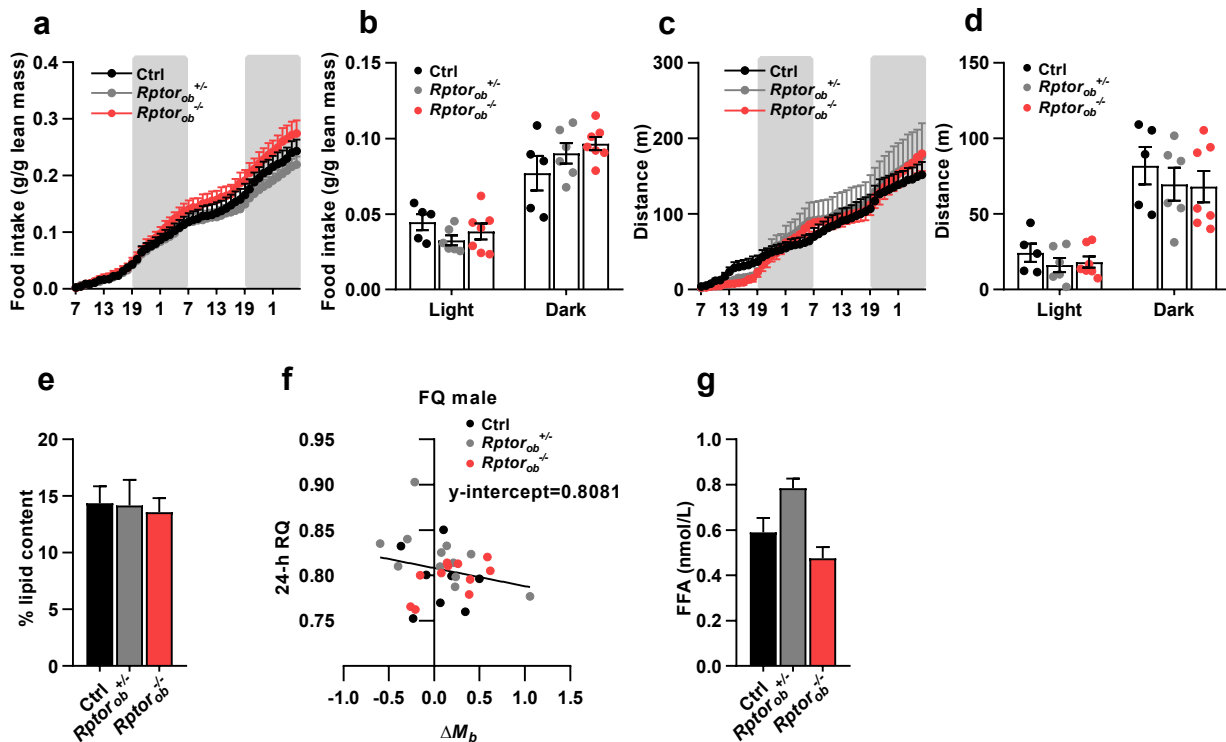

Supplement: Supplementary file 7 — Supplementary Figure 7 [file 41413_2020_123_MOESM7_ESM.pdf]

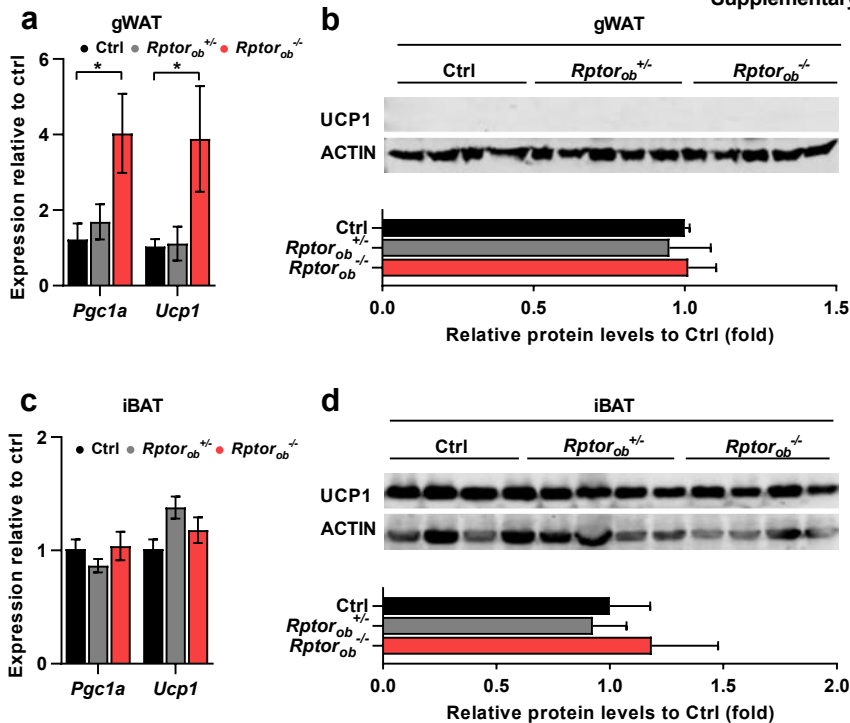

Supplement: Supplementary file 8 — Supplementary Figure 8 [file 41413_2020_123_MOESM8_ESM.pdf]

Supplementary Fig. 9

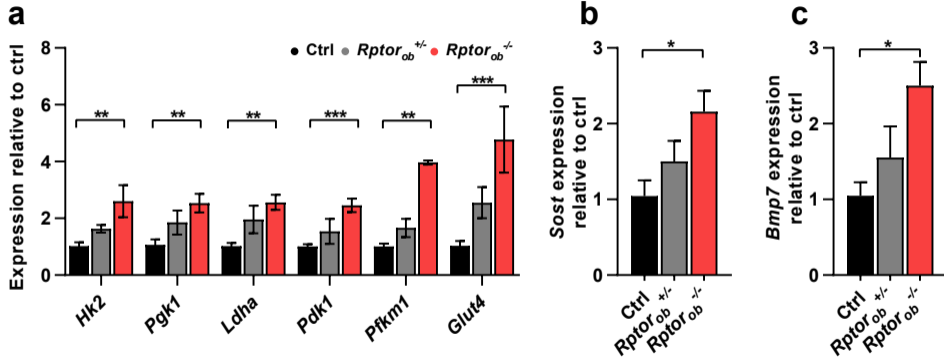

Supplement: Supplementary file 9 — Supplementary Figure 9 [file 41413_2020_123_MOESM9_ESM.pdf]

## Supplementary Fig. 10

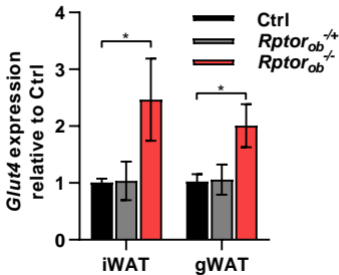

Supplement: Supplementary file 11 — Supplementary Figure 10 [file 41413_2020_123_MOESM11_ESM.pdf]
