# Supplementary material for: The mTORC1 complex in pre-osteoblasts regulates whole-body energy metabolism independently of osteocalcin
Source: Bone Res. 2021 Feb 8;9:10. doi: 10.1038/s41413-020-00123-z (PMC7868369; doi:10.1038/s41413-020-00123-z)
Supplement: Supplementary file 6 — Supplementary Figure 6 [file 41413_2020_123_MOESM6_ESM.pdf]

Supplementary Fig. 6

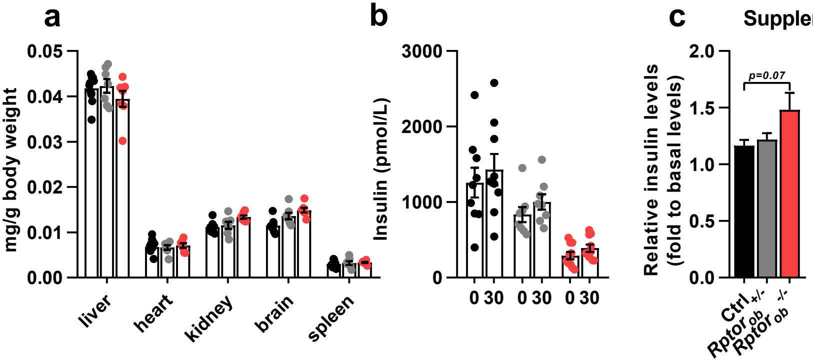

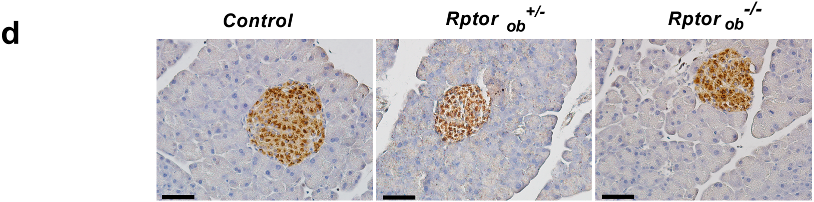

	Ctrl (n=8)	*Rptor*$_{ob}^{-/+}$ (n=5)	*Rptor*$_{ob}^{-/-}$ (n=7)
β-cell mass (mg)	1.674 ± 0.245	1.210 ± 0.263	0.856 ± 0.098*
No. of Islets per Pancreatic area	0.638 ± 0.096	0.641 ± 0.136	0.640 ± 0.056
Average Islets area ($x10^3$ μm^2)	12.560 ± 0.954	8.802 ± 1.278*	8.420 ± 0.784*
