# Supplementary material for: The mTORC1 complex in pre-osteoblasts regulates whole-body energy metabolism independently of osteocalcin
Source: Bone Res. 2021 Feb 8;9:10. doi: 10.1038/s41413-020-00123-z (PMC7868369; doi:10.1038/s41413-020-00123-z)

Fig. 3f : Adiponec n (HMW/LMW)

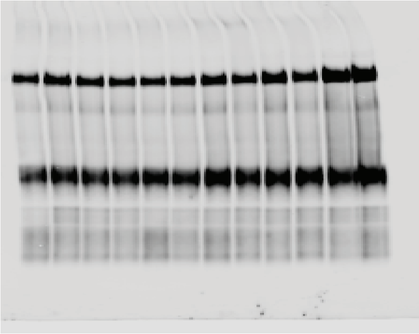

Fig. 3ef:Adiponec n (total)

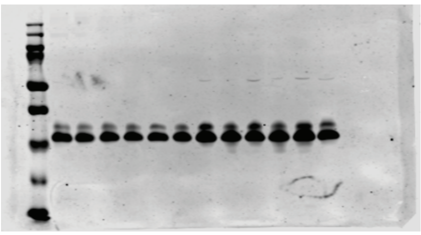

Fig. 3i : Adipoq/ac n (gWAT)

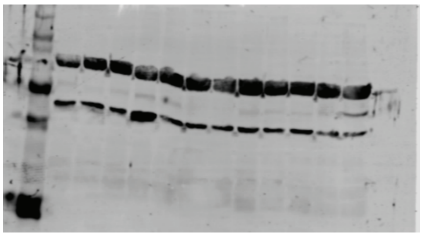

ACTIN

ADIPOQ

Fig. 3i : Adipoq/ac n (iWAT)

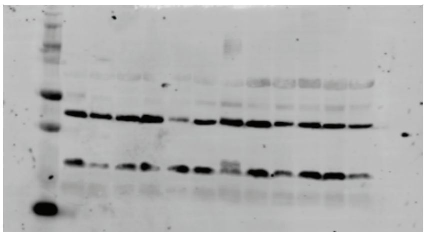

ACTIN

ADIPOQ

Fig. 3i : actin (Tib/fem)

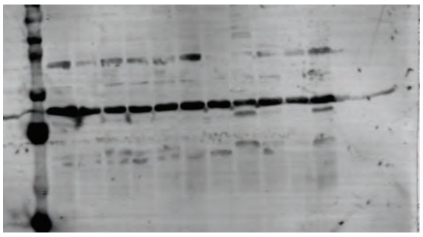

ACTIN

Fig. 3i : Adipoq (Tib/fem)_ADIPOQ

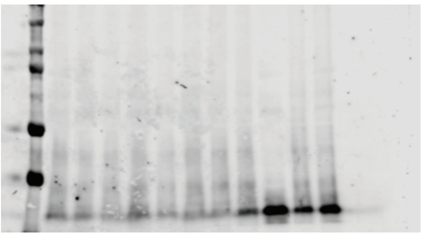

Fig. 5a:muscle

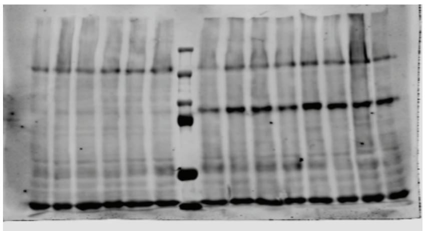

IRS1

P-INSR

ACTIN

Fig. 5a:muscle_INSR

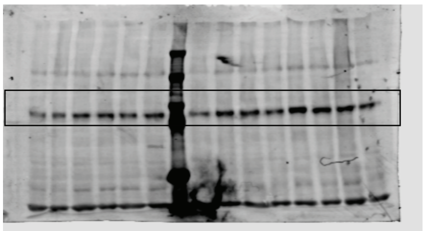

Fig. 5a:muscle_p-AKT

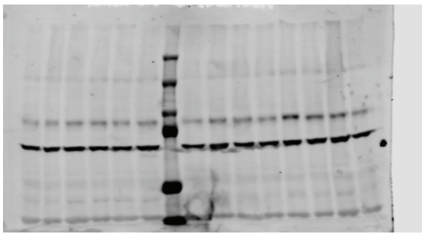

Fig. 5a:muscle_AKT

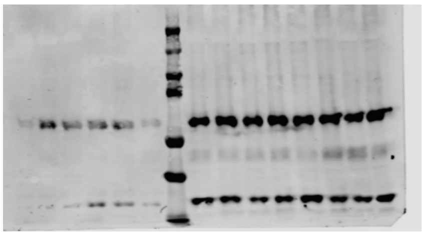

Fig. 5a:muscle_p-rpS6

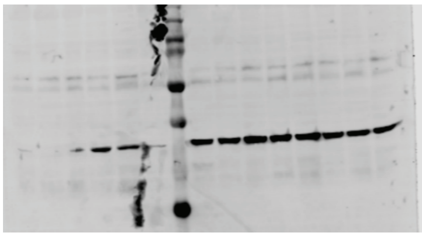

Fig. 5a:muscle_rpS6

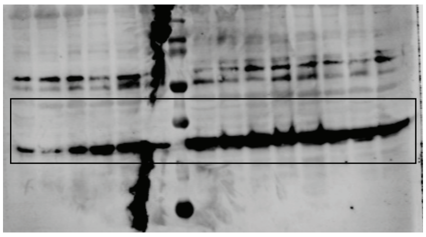

Fig. 5b:liver_IRS1

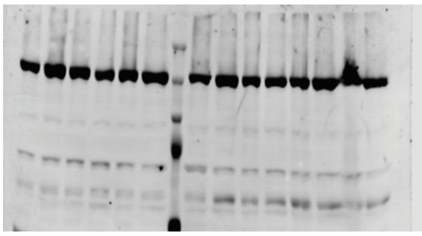

Fig. 5b:liver_ACTIN

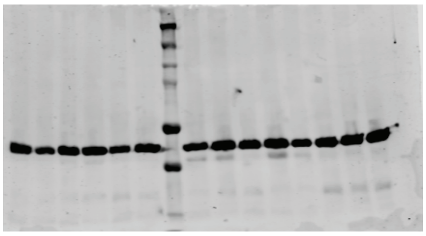

Fig. 5b:liver_p-INSR

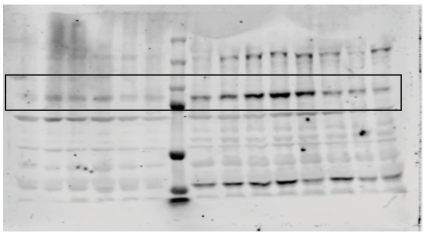

Fig. 5b:liver_INSR

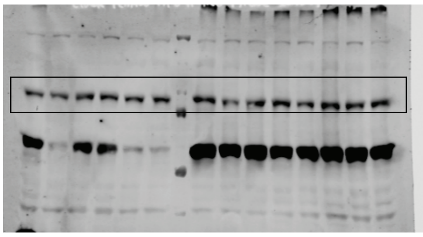

Fig. 5b:liver_p-AKT

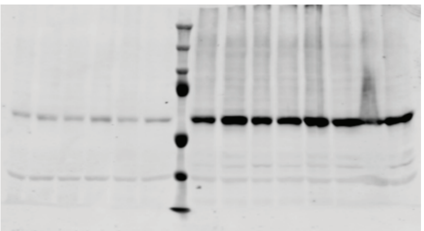

Fig. 5b:liver_AKT

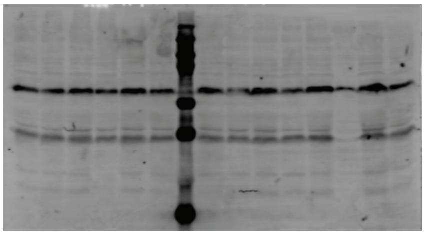

Fig. 5b:liver_p-rpS6

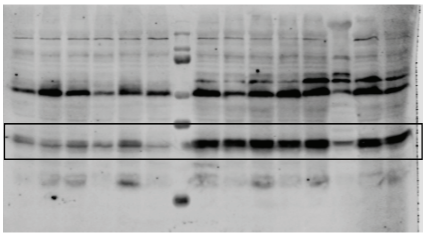

Fig. 5b:liver_S6

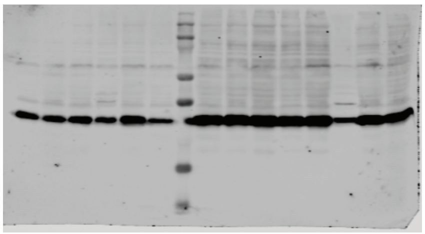

Fig. 5c:iWAT_IRS1

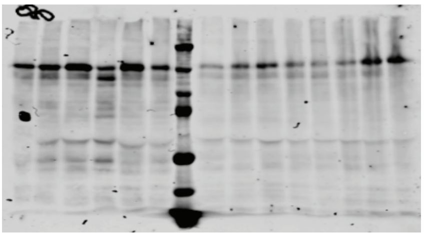

Fig. 5c:iWAT_ACTIN

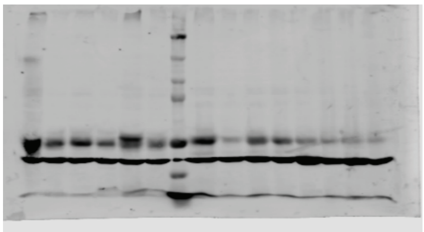

Fig. 5c:iWAT

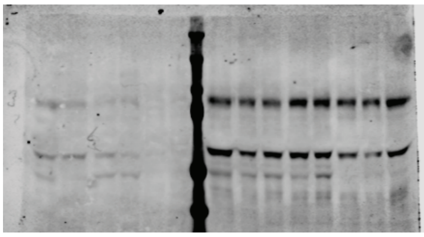

P-INSR

P-AKT

Fig. 5c:iWAT

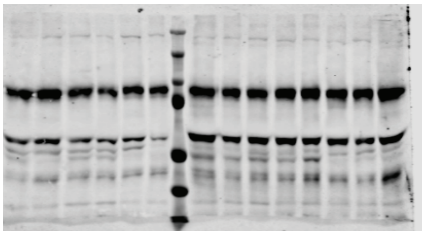

INSR

AKT

Fig. 5c:iWAT_p-rpS6

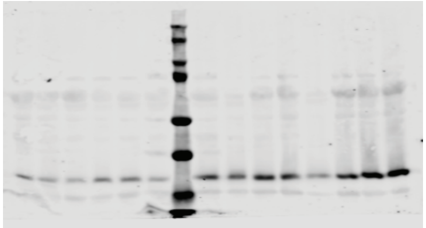

Fig. 5c:iWAT_rpS6

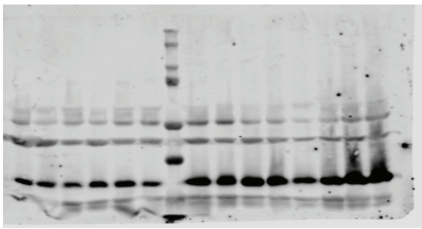

Fig. 5d:gWAT_IRS1

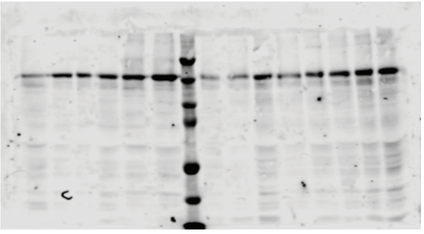

Fig. 5d:gWAT_ACTIN

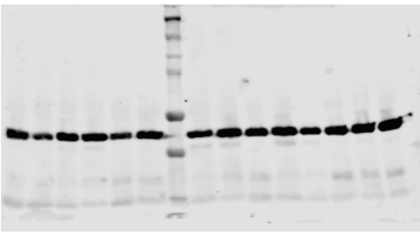

Fig. 5d:gWAT_p-INSR

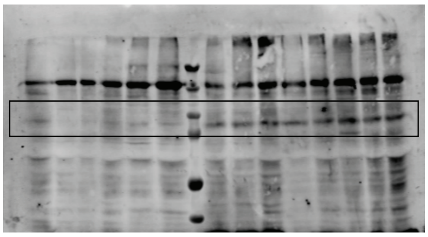

Fig. 5d:gWAT_INSR

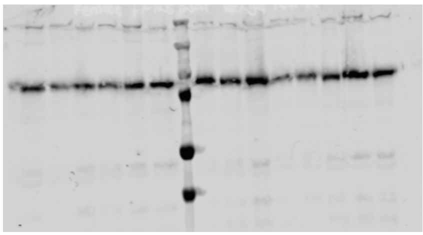

Fig. 5d:gWAT_p-AKT

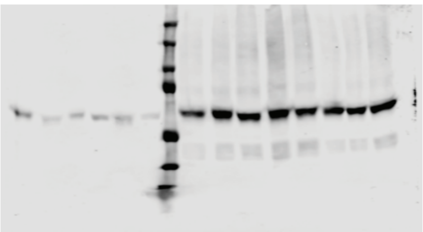

Fig. 5d:gWAT_AKT

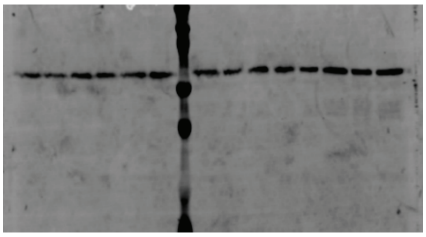

Fig. 5d:gWAT_p-rpS6

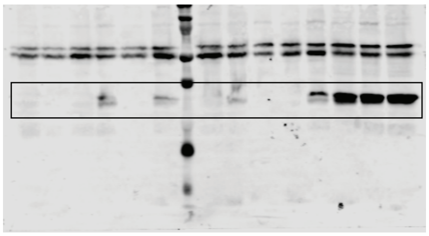

Fig. 5d:gWAT_rpS6

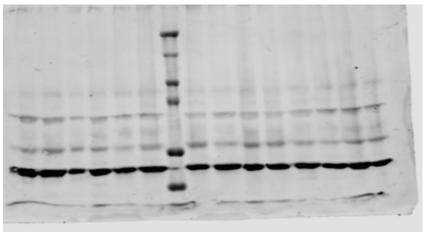

Fig. 5j: Adiponectin (HMW/LMW)

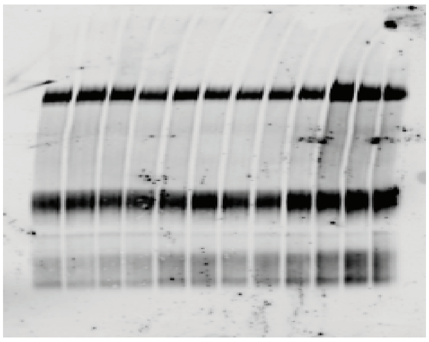

Fig. 5j: Adiponectin (total)

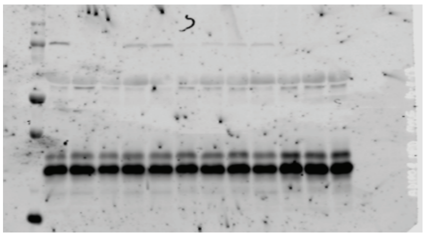

Fig. 6o: ACTIN/UCP1 (iWAT)

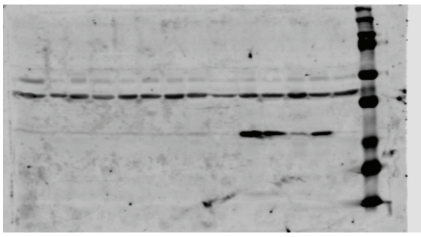

Fig. 8b: calvaria +/- insulin

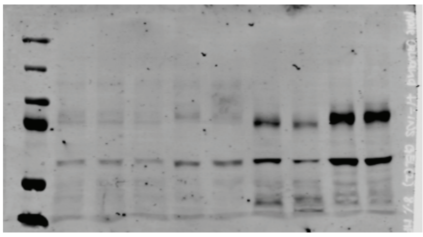

Fig. 8b: calvaria +/- insulin

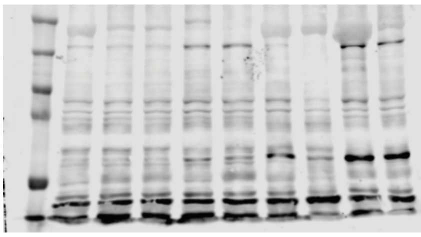

Fig. 8b: calvaria +/- insulin

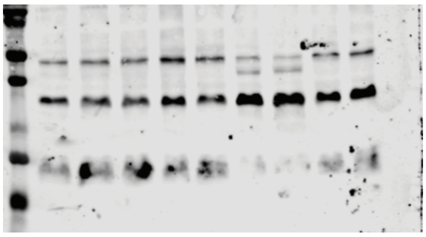

Fig. 8b: calvaria +/- insulin

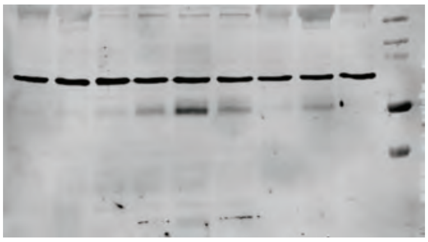

Fig. 8b: calvaria +/- insulin

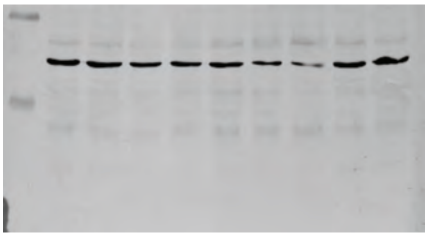

Fig. 8b: calvaria +/- insulin

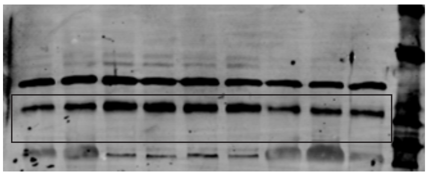

rpS6

Fig. 8b: calvaria +/- insulin

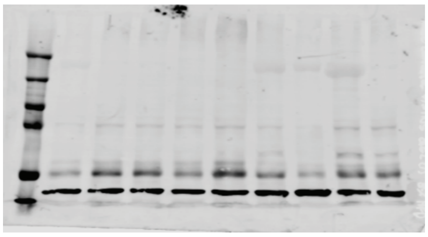

ACTIN

Fig. 8c : RAPTOR

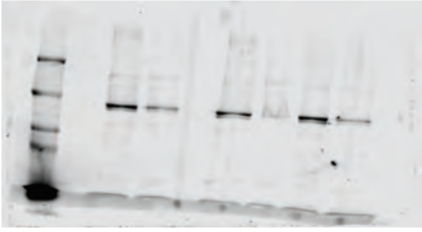

Fig. 8c : p-rpS6

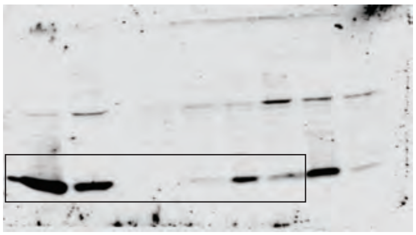

Fig. 8c : rpS6

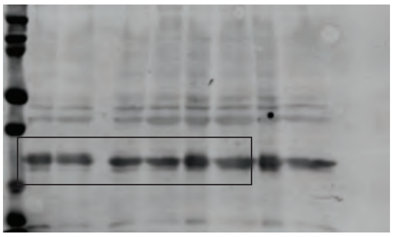

Fig. 8c : 4E-BP1

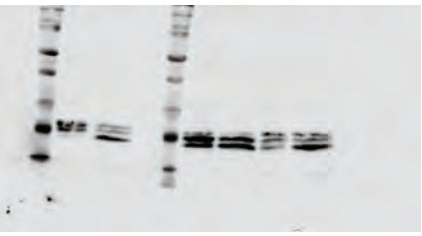

Fig. 8c : p-INSR

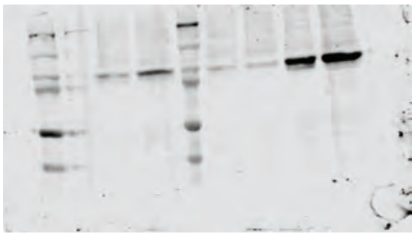

Fig. 8c : INSR

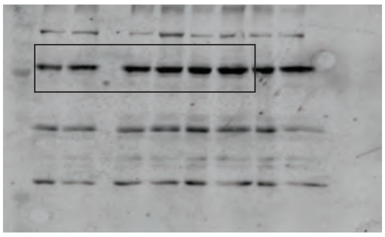

Fig. 8c : p-AKT S473

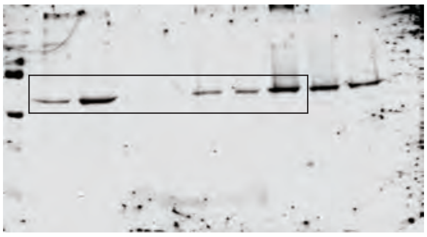

Fig. 8c : p-AKT T308

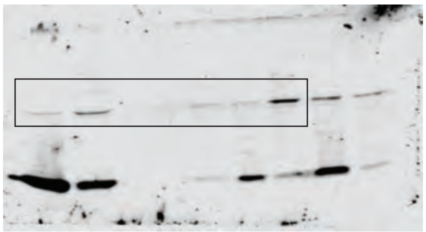

Fig. 8c : AKT

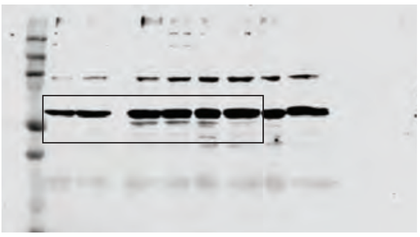

Fig. 8c : p-GSK

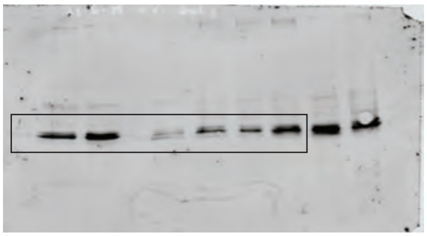

Fig. 8c : GSK

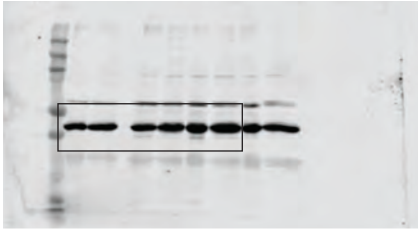

Fig. 8c : a-TUBULIN

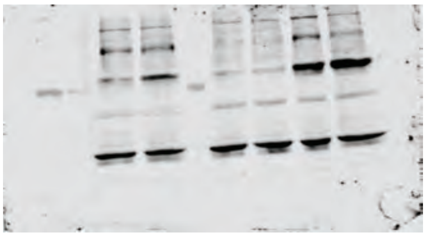

Supp Fig. 2b: iWAT_RAPTOR

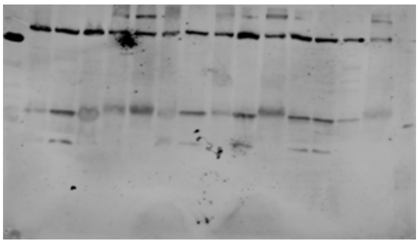

RAPTOR

Supp Fig. 2b: iWAT_actin

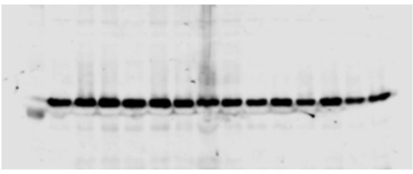

Supp Fig. 2b: liver_RAPTOR

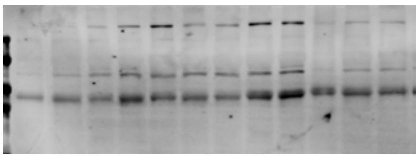

RAPTOR

Supp Fig. 2b: liver_actin

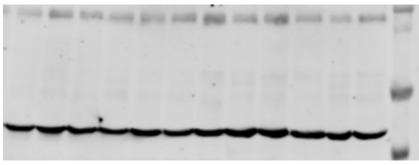

Supp Fig. 2b: muscle_RAPTOR

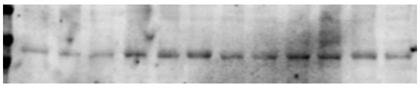

RAPTOR

Supp Fig. 2b: muscle_actin

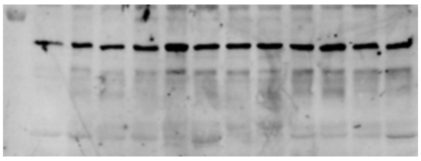

Supp Fig. 3m: iBAT_UCP1

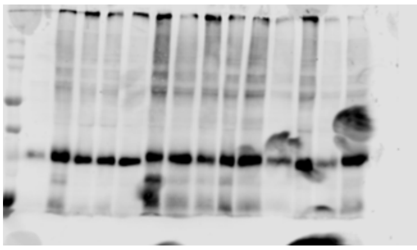

Supp Fig. 3m: iBAT_HCS70

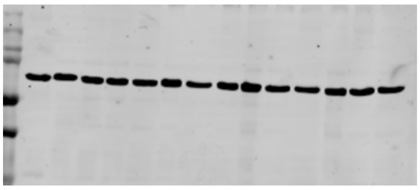

Supp Fig.4c : liver (+PBS)

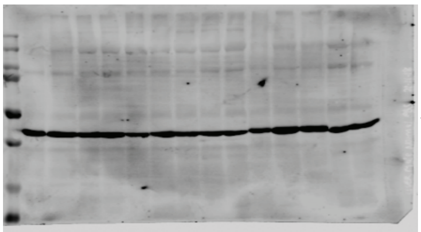

p-INSR

ACTIN

Supp Fig. 4c: liver (+insulin)

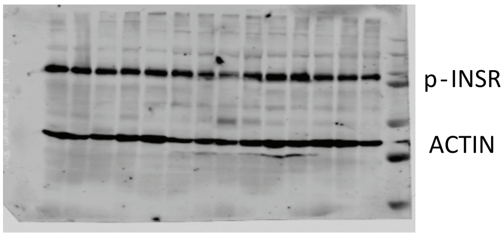

p-INSR

ACTIN

Supp Fig. 4c: liver (+PBS)_INSR

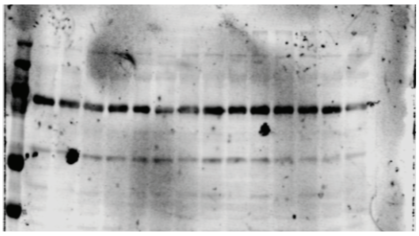

Supp Fig. 2c: liver (+PBS)_p-AKT

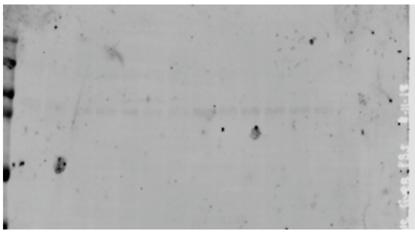

Supp Fig. 4c: liver (+insulin)_p-AKT

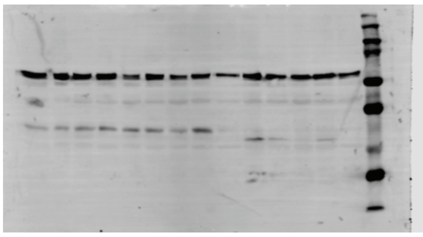

Supp Fig. 4c: liver (+insulin)_AKT

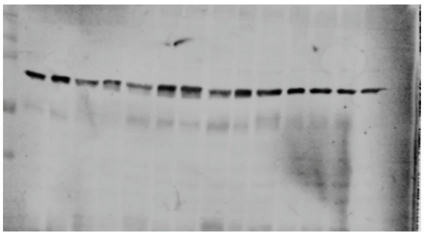

Supp Fig. 4d: muscle(+insulin)_p-INSR

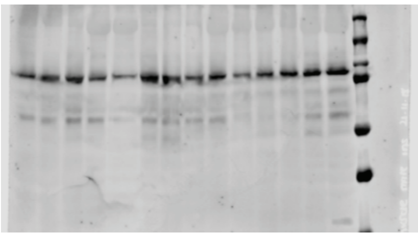

Supp Fig. 4d: muscle(+insulin)_p-AKT

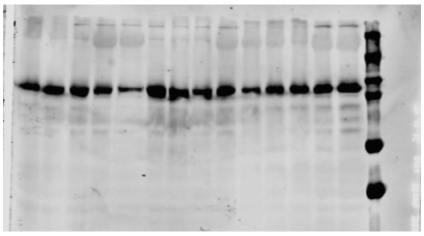

Supp Fig.8b : gWAT UCP1 and ACTIN

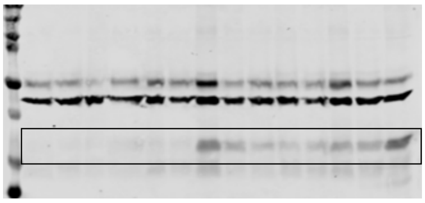

Supp Fig. 4c: liver (+PBS)_AKT

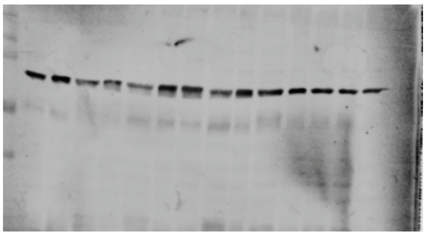

Supp Fig. 4d: muscle(+PBS)_p-INSR

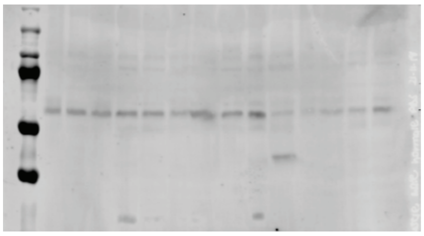

Supp Fig. 4d: muscle(+PBS)_INSR

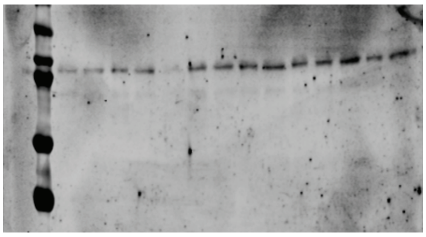

Supp Fig. 4d: muscle(+PBS)_p-AKT

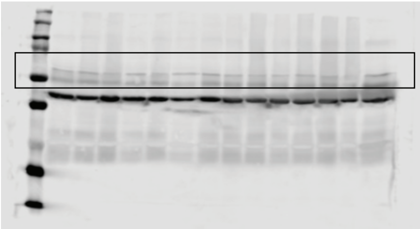

Supp Fig.8b : iBAT UCP1 and ACTIN

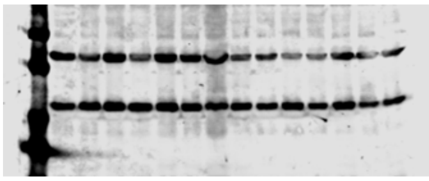

Supplement: Supplementary file 10 — Supplementary Figure 11 [file 41413_2020_123_MOESM10_ESM.pdf]
