# Supplementary material for: The mTORC1 complex in pre-osteoblasts regulates whole-body energy metabolism independently of osteocalcin
Source: Bone Res. 2021 Feb 8;9:10. doi: 10.1038/s41413-020-00123-z (PMC7868369; doi:10.1038/s41413-020-00123-z)
Supplement: Supplementary file 13 — Supplementary Table 2_final.docx [file 41413_2020_123_MOESM13_ESM.docx]

| **Supplementary Table 2.** Antibodies used for immunoblotting | | | | |
| --- | --- | --- | --- | --- |
| **Antibody** | **Species** | **Dilution** | **Company** | **Catalogue number** |
| ACTIN | Mouse | 1:1000 | Sigma-Aldrich | A5441 |
| ADIPONECTIN | Rabbit | 1:1000 | Sigma-Aldrich | A6354 |
| AKT | Rabbit | 1:1000 | Cell Signalling Technology | CST4691S |
| INSR | Rabbit | 1:1000 | Cell Signalling Technology | CST3025 |
| Phospho-AKT (Ser473) | Mouse | 1:1000 | Cell Signalling Technology | CST4060 |
| Phospho-AS160 (Thr642) | Rabbit | 1:1000 | Cell Signalling Technology | CST4288 |
| Phospho-GSK3β (Ser9) | Rabbit | 1:1000 | Cell Signalling Technology | CST9336 |
| Phospho-INSR (Tyr1150/1151) | Rabbit | 1:1000 | Cell Signalling Technology | CST3024 |
| Phospho-rpS6 (Ser240/244) | Rabbit | 1:1000 | Cell Signalling Technology | CST2215S |
| rpS6 | Mouse | 1:1000 | Cell Signalling Technology | CST2317 |
| UCP1 | Rabbit | 1:5000 | Abcam | ab209483 |
