# Supplementary material for: The mTORC1 complex in pre-osteoblasts regulates whole-body energy metabolism independently of osteocalcin
Source: Bone Res. 2021 Feb 8;9:10. doi: 10.1038/s41413-020-00123-z (PMC7868369; doi:10.1038/s41413-020-00123-z)
Supplement: Supplementary file 14 — Supplementary Table 3_final.docx [file 41413_2020_123_MOESM14_ESM.docx]

| **Supplementary Table 3.** Oligonucleotides used for Real-time PCR (qRT-PCR) | | | | |
| --- | --- | --- | --- | --- |
| **Gene** | **Accession** | **Forward (5’ – 3’)** | **Reverse (5’ – 3’)** | **Size (bp)** |
| *Acaca* | NM_133360.2 | ATGCGATCTATCCGTCGGTG | CCAGCCCACACTGCTTGT | 224 |
| *Acadm* | NM_007382.5 | TTCGAAGACGTCAGAGTGCC | CTGCGACTGTAGGTCTGGTT | 103 |
| *Acox* | NM_015729.3 | CATGTGGTTTAAAAACTCTGTGC | GGCATGAAGAAACGCTCCTG | 123 |
| *Acly* | NM_001199296.1 | TTCCTCCTTAATGCCAGCGG | GACTTGGGACTGAATCTTGGGG | 136 |
| *Actin* | NM_007393.5 | TTGCTGACACGATGCAGGA | AAGGGTGTAAAACGCAGCTC | 236 |
| *Adipoq* | NM_009605.4 | GATGCAGGTCTTCTTGGTCCTA | AGCGAATGGGTACATTGGGA | 187 |
| *Atgl* | NM_025802.3 | CAACGCCACTCACATCTACGG | TCACCAGGTTGAAGGAGGGAT | 161 |
| *Bglap* | NM_001032298.3 | AAGCGAGAGGGCAATAAGGT | TCAAGCCATACTGGTCTGATAGC | 142 |
| *Cidea* | NM_007702.2 | CAGTTCCTGGTCTATGCGGG | AACCAGCCTTTGGTGCTAGG | 92 |
| *Cox7a* | NM_009944.3 | AGACTGACCATGACGCTGAC | GTGTCACTTCTTGTGGGGGAA | 87 |
| *Cyp11α* | NM_019779.4 | CCTTTCCTGAGCCCTACGTG | GCCCAGCTTCTCCCTGTAAA | 222 |
| *Fasn* | NM_007988.3 | TGGGTGTGGAAGTTCGTCAG | CTGTCGTGTCAGTAGCCGAG | 132 |
| *Foxa2* | NM_001291065.1 | CACTCGGCTTCCAGTATGC | GTTCATGCCATTCATCCCCAG | 138 |
| *G6p* | NM_008061.4 | TGAGACCGGACCAGGAAGTC | GCAAGGTAGATCCGGGACAG | 195 |
| *Gck* | NM_010292.5 | CTGTTAGCAGGATGGCAGCTT | TTTCCTGGAGAGATGCTGTGG | 82 |
| *Glut1* | NM_011400.3 | GCTACGGGGTCTTAAGTGCG | CCTCCCACAGCCAACATGAG | 169 |
| *Glut4* | NM_009204.2 | GGCTCTGACGATGGGGAAC | GCCACGTTGCATTGTAGCTC | 157 |
| *Hk2* | NM_013820.3 | TGATCGCCTGCTTATTCACGG | AACCGCCTAGAAATCTCCAGA | 112 |
| *Lpl* | NM_008509.2 | GGACGGTAACGGGAATGTATG | ACGTTGTCTAGGGGGTACTTAAA | 214 |
| *Nrf1* | NM_001164226.1 | ATCTGGCTGCTGCAGGTCC | CACTCGCGTCGTGTACTCAT | 170 |
| *Pcg-1α* | NM_008904.2 | TGTGTGCTGTGTGTCAGAGT | ACCAGAGCAGCACACTCTATG | 126 |
| *Pdk4* | NM_013743.2 | GATTGACATCCTGCCTGACC | CATGGAACTCCACCAAATCC | 98 |
| *Pepck* | NM_011044.3 | ATCATCTTTGGTGGCCGTAG | TGATGATCTTGCCCTTGTGT | 136 |
| *Pfkm1* | NM_001163487.1 | GGTTTCCCTGTACTCCATGC | TTCATCACTGTGGTGCGAGT | 251 |
| *Pgk1* | NM_008828.3 | TTTGGACAAGCTGGACGTGA | AGCAGCCTTGATCCTTTGGTT | 106 |
| *Pparγ* | NM_001127330.2 | TTTTCGGAAGAACCATCCGATT | ATGGCATTGTGAGACATCCCG | 139 |
| *Rptor* | NM_028898.2 | TGGGTCTTCAACAAGAACTACACT | TCTGGGCAAGTGGATGGTTT | 199 |
| *Srebf1* | NM_001313979.1 | GTGGGCCTAGTCCGAAGC | GAGCATGTCTTCGATGTCGTT | 135 |
| *Ucp1* | NM_009463.3 | TGGTGAACCCGACAACTTCC | GGCCTTCACCTTGGATCTGAA | 141 |
| *Ucp2* | NM_011671.5 | TGCGGTCCGGACACAATAGTA | CTCGTTCTTCAAAGCTGCCG | 111 |
